# Protein phosphatase PP2Cα S-glutathionylation regulates cell migration

**DOI:** 10.1016/j.jbc.2024.107784

**Published:** 2024-09-18

**Authors:** Dhanushika S.K. Kukulage, Kusal T.G. Samarasinghe, Nadee N.J. Matarage Don, Madhu C. Shivamadhu, Kyosuke Shishikura, William Schiff, Faezeh Mashhadi Ramezani, Rayavarapu Padmavathi, Megan L. Matthews, Young-Hoon Ahn

**Affiliations:** 1 Department of Chemistry, Drexel University, Philadelphia, Pennsylvania, USA; 2 Amgen Inc., Thousand Oaks, California, USA; 3 Department of Chemistry, University of Pennsylvania, Philadelphia, Pennsylvania, USA

**Keywords:** PP2Cα phosphatase, glutathionylation, redox signaling, cell migration, reactive oxygen species, epidermal growth factor

## Abstract

Redox signaling is a fundamental mechanism that controls all major biological processes partly *via* protein cysteine oxidations, including S-glutathionylation. Despite over 2000 cysteines identified to form S-glutathionylation in databases, the identification of redox cysteines functionally linked to a biological process of interest remains challenging. Here, we demonstrate a strategy combining glutathionylation proteomic database, bioinformatics, and biological screening, which resulted in the identification of S-glutathionylated proteins, including PP2Cα, as redox players of cell migration. We showed that PP2Cα, a prototypical magnesium-dependent serine/threonine phosphatase, is susceptible to S-glutathionylation selectively at nonconserved C314. PP2Cα glutathionylation causes increased migration and invasion of breast cancer cell lines in oxidative stress or upon hydrogen peroxide production. Mechanistically, PP2Cα glutathionylation modulates its protein–protein interactions, activating c-Jun N-terminal kinase and extracellular signal-regulated kinase pathways to elevate migration and invasion. In addition, PP2Cα glutathionylation occurs in response to epidermal growth factor, supporting a serine/threonine phosphatase PP2Cα as a new redox player in growth factor signal transduction.

Cell migration is a central process in the development, organization, and maintenance of multicellular organisms, as evidenced in embryogenesis, wound healing, and immune response (1), while its dysregulation is a major event in pathologies, including cancer metastasis and inflammatory diseases (1, 2, 3). Cells migrate in response to diverse signals, including chemokine and physical cues (4). Importantly, numerous pieces of evidence support that reactive oxygen species (ROS), such as hydrogen peroxide (H_2_O_2_), play a central role in cell migration (5). For example, H_2_O_2_ locally accumulates at a leading edge and protrusions of cells, inducing cell polarization and migration (6). H_2_O_2_ is produced at the wound margin, acting as a chemokine that directs the migration of leukocytes to the wound (7). Similarly, expressions of NADPH oxidases and other oxidases, which produce ROS or H_2_O_2_, induce the migration of various cell types, including epithelial cells, fibroblasts, and endothelial cells (8, 9, 10, 11, 12). Moreover, oxidative stress and ROS were found to increase motility and invasion of cancer cells and regulate epithelial-mesenchymal transition (EMT) (13, 14, 15, 16).

In agreement, extensive mechanistic studies have delineated proteins and signaling pathways modulated by ROS in regulating cell migration (17). For example, ROS activate mitogen-activated protein kinases (MAPK) *via* activation of receptor tyrosine kinase (18, 19), inactivation of protein tyrosine phosphatase (PTP) (20), and activation of GTPase (*e.g.*, Rac1) (21). Similarly, ROS induce the activation of focal adhesion kinase and other kinases (*e.g.*, Src and PKC) (13). These signaling pathways collectively remodel actin filaments and cytoskeletal structure, serving as the primary mechanisms for cell migration and adhesion (17).

It is now well-established that ROS control redox signaling *via* diverse protein cysteine oxidations, including S-sulfenylation, disulfide (-SS-), S-sulfenamide, S-glutathionylation (SSG), and a recently discovered covalent nitrogen-oxygen-sulfur bridge (22, 23). Evidence supports that functionally distinct protein networks and biological processes are modulated by individual cysteine oxoforms that feature unique sizes, reactivities, and stabilities (24). Among diverse cysteine oxidations, protein S-glutathionylation is one of the major cysteine oxidations forming in response to ROS or oxidative stress (25). The biological significance of protein S-glutathionylation has been demonstrated in all areas of health and diseases (26). Accordingly, previous studies have shown that S-glutathionylation of specific target proteins, such as MAPK phosphatase-1 (MKP-1) (27), 14-3-3 zeta (28), low molecular weight protein tyrosine phosphatase (LMW-PTP) (29), and actin (30), regulate cell migration and adhesion. We also recently demonstrated that p120 catenin glutathionylation increases epithelial cell migration and invasion *via* E-cadherin destabilization (31), thus exemplifying S-glutathionylation in cell migration. Despite these examples, the identification of proteins regulating cell motility, specifically *via* S-glutathionylation, remains limited.

In this report, we established an integrative platform combining chemical proteomic data, bioinformatics, and biological screening (*i.e.*, cell migration assay), which systematically led us to discover glutathionylation of three new proteins, including protein phosphatase 2C isoform α (PP2Cα, also known as PPM1A), that regulate cell migration. PP2Cα is a metal (Mg^2+^/Mn^2+^)-dependent Ser/Thr phosphatase, which catalyzes the dephosphorylation of its substrates by a magnesium-coordinated water molecule. Unlike PTPs, PP2Cα does not harbor an oxidation-prone reactive cysteine in the active site (32). As a Ser/Thr phosphatase, PP2Cα regulates diverse biological processes, especially stress signaling. For example, PP2Cα negatively regulates cell proliferation, migration, and mitogen-signaling (MAPK, including c-Jun N-terminal kinase (JNK) and p38) (33, 34), metabolism (*e.g.*, AMPK) (35), cell cycle (CDK2 and CDK6), TGF-β signaling (SMAD2 and SMAD3) (36), inflammation (IKKβ and p65) (37, 38), and innate immunity (STING and TBK1) (39, 40). Accordingly, PP2Cα knockdown increased cell proliferation, migration, invasion, and EMT (41). PP2Cα depletion was frequently seen in estrogen-receptor negative breast cancers, including metastatic triple-negative breast cancers (42). In addition to cancers, PP2Cα is implicated in fibrosis, diabetes, infectious diseases, and neurodegeneration (41).

In this report, we show that PP2Cα is susceptible to glutathionylation selectively at Cys 314 (C314) located at the C-terminal domain (CTD), whose function remains largely unknown. We further showed that PP2Cα C314 glutathionylation increases migration and invasion of MDA-MB-231 cells under oxidative stress, such as glucose depletion, *via* selective activation of JNK and extracellular signal-regulated kinase (ERK) pathways. In addition, we demonstrate that PP2Cα is susceptible to glutathionylation in response to epithelial growth factor (EGF), which increases epithelial MCF7 cell migration. These findings suggest that PP2Cα glutathionylation constitutes a prototypical growth factor redox signaling event.

## Result

### Identification of redox regulatory proteins and cysteines for cell migration

**Figure 1:**
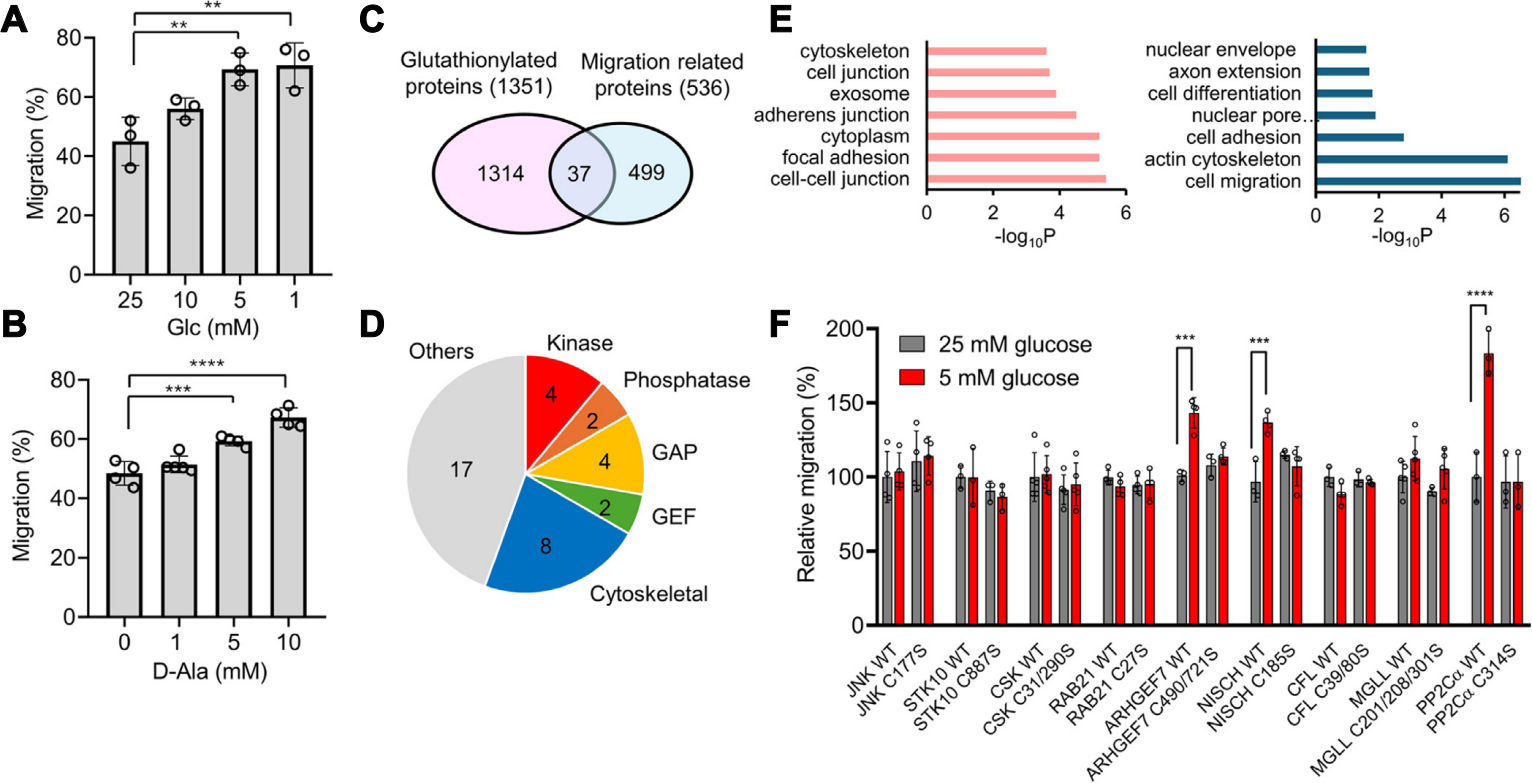
**A strategy to identify functional cysteines regulating cell migration *via* S-glutathionylation.** Proteomic data, bioinformatics, and biological screening (*i.e.*, cell migration) were combined to narrow down regulatory cysteines for cell migration. A–*B*, low glucose or D-amino acid oxidase (DAAO)-induced H_2_O_2_ increases cell migration. MCF7 cells were incubated with decreasing concentrations of glucose (n = 3) (*A*) or MCF7 cells expressing DAAO were incubated with increasing concentrations of D-Ala for 24 h (n = 4) (*B*). *C,* Cytoscape bioinformatic analysis comparing glutathionylated proteins and migration-related proteins. *D*, functional categories of 37 proteins belonging to “S-glutathionylation” and “migration” groups, including GTPase-activating protein (GAP) and guanine nucleotide exchange factor (GEF). *E*, gene ontology (GO) analysis of the identified 37 proteins. *F*, cell migration screening of nine selected proteins. MDA-MB-231 cells expressing protein WT or cysteine-to-serine mutant (C/S) were subjected to high or low glucose conditions. The migration was measured using the wound-healing assay and normalized by the average migration levels of each protein WT at 25 mM glucose (n = 3). ∗STK10, Rab21, and MGLL with cysteine numbers are from mouse, and all others are from human. Data represent the mean ± SD. The statistical difference was analyzed by one-way ANOVA and Tukey’s *post hoc* test (*A*, *B*) or two-way ANOVA followed by Tukey’s *post hoc* test (*F*), where ∗*p* < 0.03, ∗∗*p* < 0.002, ∗∗∗*p* < 0.0002, ∗∗∗∗*p* < 0.0001.

To demonstrate enhanced cell migration by ROS or oxidative stress, we analyzed the migration of breast cancer cell lines (MCF7 and MDA-MB-231) upon induction of ROS or H_2_O_2_. Previously, we have shown that low glucose, compared to high glucose concentrations, increase ROS and induce global glutathionylation in various cell lines (31, 43, 44). Similarly, lower glucose (5 and 1 mM) compared to higher glucose (25 mM) increased ROS in MCF7 and MDA-MB-231 cells (Fig. S1*A*). The low glucose may represent a condition of glucose-deprived or physically confined tissue or solid tumor with disorganized vasculature (45, 46), thus adapting cells to migrate for metabolic balance. The wound-healing migration assays showed that MCF7 increased cell migration with lower glucose (5 and 1 mM) than higher glucose conditions (25 mM) (Figs. 1*A* and S1*B*). To support ROS or H_2_O_2_-mediated migration, we overexpressed D-amino acid oxidase (DAAO) (47) to MCF7 in which increasing concentrations of D-Ala (0–10 mM) caused a concentration-dependent increase of cell migration (Figs. 1*B* and S1*C*). The increased cell migration by D-Ala was not observed in MCF7 without expressing DAAO (Fig. S1*D*). Similarly, MDA-MB-231 showed increased migration under similar conditions (1 mM *versus* 25 mM glucose), albeit a less significant increase (Fig. S1*E*). However, we did not observe increased migration of MCF7 cells upon a bolus addition of H_2_O_2_ (0–100 μM) (Fig. S1*F*), suggesting that intracellular H_2_O_2_ production may be necessary for increased cell migration.

Next, we sought to identify redox cysteines regulating cell migration *via* glutathionylation. Despite many proteomic studies, a systematic or unbiased approach for discovering redox-regulatory cysteines functionally linked to a biological process of interest (*e.g.*, cell migration) remains limited. That is, although proteomic analyses have identified a number of cysteines susceptible to S-glutathionylation (n > 2000) (26), the challenge exists in how to discover which ones out of over 2000 glutathionylation-susceptible cysteines regulate a biological process of interest. To address the challenge, we established a systematic platform that integrates proteomics, bioinformatics, and biological screening, which enabled the identification of new regulatory cysteines for cell migration. Our strategy involves two steps: first, we used proteomic data and bioinformatic analysis to narrow down glutathionylation-susceptible cysteines associated with cell migration (Fig. 1*C*). These analyses were conducted using a database of glutathionylated proteins (n = 1351) established with our clickable glutathione approach (Table S1) (44, 48, 49). Importantly, unlike other existing approaches (26), the clickable glutathione approach coupled with mass spectrometry (*i.e.*, LC-MS/MS) detects glutathionylated peptides with a glutathione modification (Fig. S2). Therefore, the approach unequivocally detects glutathionylated cysteine sites, allowing us to select candidate cysteines for biological screenings. The database of glutathionylated proteins (n = 1351 for proteins; n = 2124 for cysteines, Table S1) was compared with a list of proteins associated with cell migration (n = 536, Table S1) in Cytoscape analysis, which resulted in 37 proteins both susceptible to glutathionylation and associated with cell migration (Figs. 1*C* and S3*A* , and Table S1). The list of 37 proteins includes kinase (10.8%, *e.g.*, JNK and CSK), phosphatase (5.4%, *e.g.*, PP2Cα), GTPase regulators (16.2%, *e.g.*, ARHGEF7), and cytoskeletal proteins (21.6%, *e.g.*, CFL1) (Figs. 1*D* and Fig. S3*A*). The gene ontology (GO) analysis of 37 proteins indicates their localizations at cell-cell junction, focal adhesion, and cytoplasm (Fig. 1*E*, left) while showing their roles in cell migration, regulation of actin cytoskeleton, and cell adhesion (Fig. 1*E*, right). The cluster analysis with their interacting proteins suggests their roles in cell-matrix adhesion and GTPase signaling (Fig. S3*B*), suggesting that glutathionylation may regulate cell–cell or cell–matrix interactions *via* GTPase- and kinase-signaling. The second step of our strategy was to systematically analyze and screen the selected candidate cysteines (n = 74 cysteines in 37 proteins) in a biological assay of cell migration. To begin with, we selected a group of cysteines (n = 14 cysteines in nine proteins) based on classifications, such as kinase, phosphatase, and GTPase signaling (Fig. 1*F* and Table S1) or availability of their plasmids. Their WT proteins and Cys-to-Ser (C/S) mutants were overexpressed (Fig. S4*A*) in MDA-MB-231 cells, which were evaluated for their relative cell migrations in the wound-healing assay under low (5 mM) or high (25 mM) glucose conditions (Figs. 1*F* and S4*B*). The wound-healing assay indicates that WT and C/S mutants of six proteins did not cause a significant difference in cell migration in high and low glucose (Fig. 1*F*). However, three WT proteins (PP2Cα, ARHGEF7, and NISCH) showed enhanced cell migration in low glucose compared to high glucose conditions (Figs. 1*F* and S4*B*). Notably, their C/S mutants (PP2Cα C314S, ARHGEF7 C490S/C721S, and NISCH C185S) negate such increases (Figs. 1*F* and S4*B*), supporting that their cysteine glutathionylation or oxidation contribute to enhanced cell migration under oxidative stress. The increased migration in cells expressing WT of three proteins, compared to their C/S mutants, were also observed in MCF7 cells (Fig. S5). Therefore, our strategy combining proteomic and bioinformatic analyses with a biological screening demonstrates a systematic platform to uncover potential regulatory cysteines for cell migration.

### PP2Cα is susceptible to glutathionylation at nonconserved Cys 314

**Figure 2:**
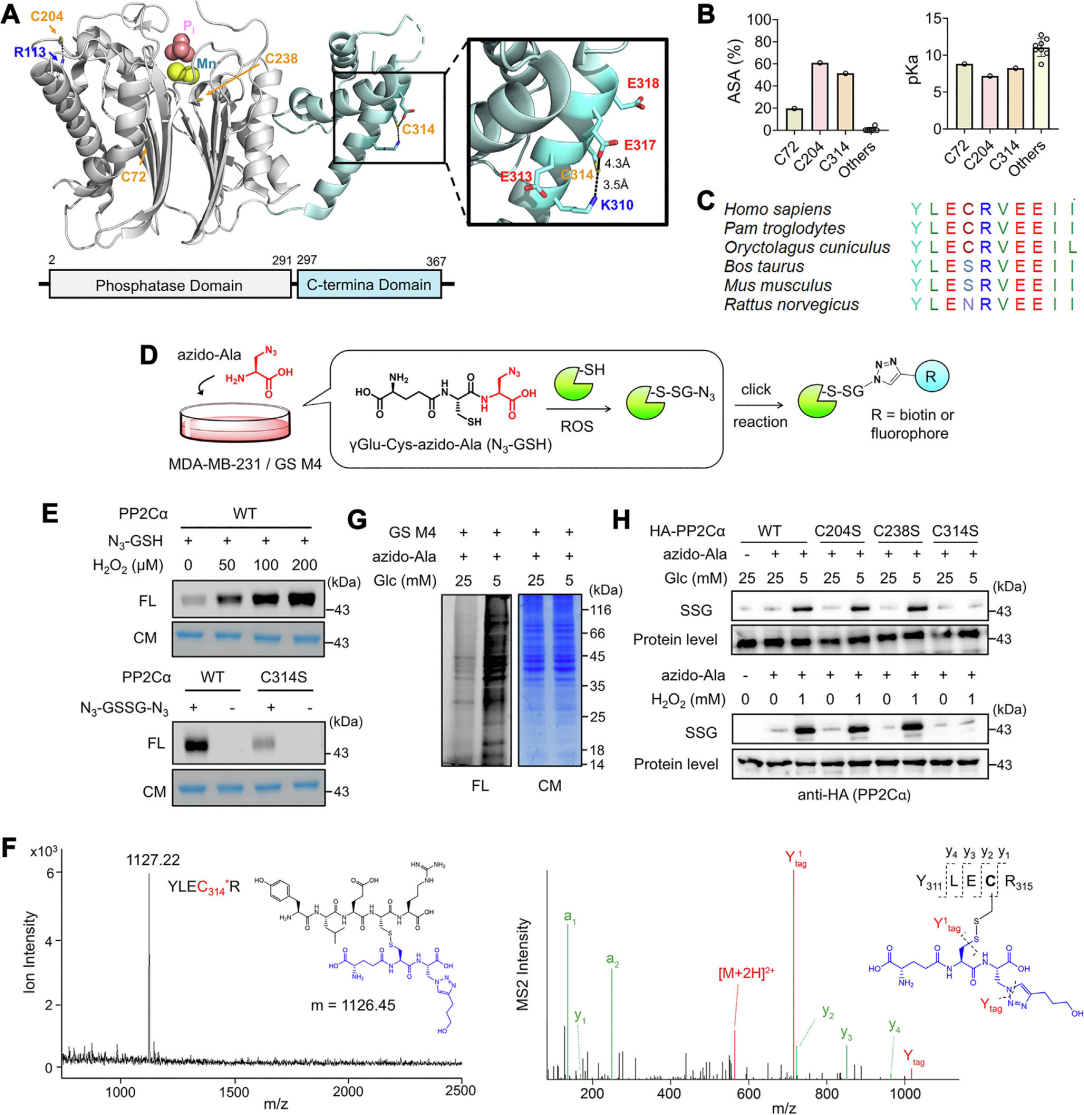
**PP2Cα is susceptible to glutathionylation at non-conserved Cys 314.***A*, PP2Cα structure. A model shows phosphate (P_i_) and Mn^2+^ in the active site and locations of three relatively surface-exposed cysteines (C72, C204, and C314). *B*, comparisons of pK_a_ and accessible surface area (ASA) for cysteines in PP2Cα. *C*, the amino acid sequence alignment around C314 among different species, showing limited conservation of C314. *D*, a scheme of a clickable glutathione approach for analyzing S-glutathionylation. Cells expressing GS M4 are incubated with azido-Ala, which enables biosynthesis of clickable glutathione (azido-glutathione, N_3_-GSH). N_3_-GSH forms S-glutathionylation in oxidative stress. Alternatively, purified protein can form glutathionylation in the presence of N_3_-GSH, which is analyzed after the click reaction. *E*, glutathionylation of purified PP2Cα *in vitro*. Purified PP2Cα was incubated with N_3_-GSH and H_2_O_2_ for 15 min (*top*) or oxidized azido-glutathione (N_3_-GSSG-N_3_) for 30 min (*bottom*). PP2Cα glutathionylated by azido-glutathione was conjugated with rhodamine-alkyne *via* click chemistry and visualized by Coomassie stain (CM, protein level) and fluorescence (FL, SSG level) (n = 3). *F*, MALDI-TOF and MS/MS analyses of glutathionylated peptides in PP2Cα. Purified PP2Cα glutathionylated by azido-glutathione was click-conjugated by biotin-DADPS-alkyne, enriched by streptavidin-agarose, and digested by trypsin on beads. Glutathionylated peptides were eluted and analyzed by MALDI-TOF (*left*) and LC-MS/MS (*right*), finding a glutathionylated peptide at C314 (YLEC_314_∗R, m/z 1127). In the MS2 spectrum, all y ions were found with additional ions (Y^1^_tag_ and Y_tag_) resulting from fragmentation in glutathione modification (n = 3). *G*, global glutathionylation in MDA-MB-231. MDA-MB-231 cells expressing GS M4 (MDA-MB-231/GS M4) were stimulated in low or high glucose conditions. After the click reaction of lysates with rhodamine-alkyne, proteins were analyzed by fluorescence and Coomassie stain (n = 3). *H*, PP2Cα glutathionylation in MDA-MB-231. MDA-MB-231/GS M4 cells expressing HA-PP2Cα WT or C/S mutant were incubated in low or high glucose conditions for 24 h or with H_2_O_2_ for 15 min. After the click reaction of lysates with biotin-alkyne, glutathionylated PP2Cα was probed by Western blot before (protein level) and after (SSG) enrichment by streptavidin-agarose (n = 3). Data are representative of three independent experiments.

Out of three proteins (PP2Cα, ARHGEF7, and NISCH), PP2Cα with C314 was selected for further investigation due to a more significant increase in cell migration than other two proteins (Fig. 1*F*). PP2Cα has 11 cysteines in two domains, a PPM-catalytic domain (CatD, amino acid 1–291) and a CTD (amino acid 297–369), where C314 is found in CTD (Figs. 2*A* and S6*A*). The available crystal structure (PDB 4RA2, human PP2Cα, Figs. 2*A* and S6*A*) predicted three cysteines (C72, C204, and C314) with the highest accessible surface area (ASA) (19.7%, 60.9%, 51.4%, respectively) (Fig. 2*B*, left). The ProPKa analysis (50) suggested their reduced pK_a_ values (8.81, 7.18, and 8.23 for C72, C204, and C314) compared to others (average pK_a_ of other eight cysteines = 11.0) (Fig. 2*B*, right), which is likely attributed to basic residues interacting with C204 and C314 (Figs. 2*A* and S6*A*). Therefore, C204 and C314 have favorable features (high ASA and low pK_a_) for cysteine oxidations (22, 24). Interestingly, C204 is highly conserved among species (Fig. S6*B*), whereas C314 is only found in human (Homo Sapiens), chimpanzee (Pam Troglodytes), and rabbit (Oryctolagus Cuniculus) but not conserved in other species, including cow (Bos Taurus) and rodents (Mus Musculus and Rattus Norvegicus) (Figs. 2*C* and S6*B*). In addition, C314 is not conserved in a close paralog, PP2Cβ (PPM1b) (Fig. S6*C*).

To demonstrate glutathionylation at PP2Cα C314, we purified WT and C314S mutant proteins of PP2Cα (Fig. S7*A*) and examined their glutathionylation with azido-glutathione synthesized *in vitro* (Fig. S7*B*). When treated with H_2_O_2_, purified PP2Cα WT showed dose-dependent glutathionylation signals after click reaction with rhodamine-alkyne (Fig. 2*E*, top, and S7C-D). Similarly, PP2Cα WT showed a strong signal for glutathionylation when incubated with oxidized azido-glutathione (N_3_-GSSG-N_3_) (Fig. 2*E*, bottom), whereas PP2Cα C314S displayed a reduced signal for glutathionylation, albeit with the remaining intensity of glutathionylation (Fig. 2*E*, bottom, and S7D), suggesting that C314 is susceptible to glutathionylation *in vitro*, potentially with other cysteines. To confirm the glutathionylated site(s), glutathionylated PP2Cα was analyzed by mass spectrometry. PP2Cα glutathionylated by azido-glutathione was conjugated with biotin-DADPS-alkyne and digested by trypsin. Biotinylated peptides were enriched by streptavidin and eluted (Fig. S2*B*). The MALDI analysis found one main peak that matches the molecular weight of a peptide glutathionylated at C314 (m/z 1127, YLEC_314_∗R) (Fig. 2*F*, left, and S8A). The subsequent LC-MS/MS analysis confirmed glutathionylation at C314 (Fig. 2*F*, right, and S8B) while also finding glutathionylation at C204 (Fig. S8*C*). In contrast, similar conditions did not yield evidence of forming sulfinic acid, sulfonic acid, or over-oxidation forms of cysteine or intermolecular disulfide formation (Fig. S7*F*).

Next, PP2Cα glutathionylation was further analyzed in MDA-MB-231 cells expressing GS M4, which synthesizes clickable glutathione in cells (Fig. 2*D*). Low glucose (5 mM) compared to high glucose (25 mM) induced a high level of global glutathionylation in MDA-MB-231 (Fig. 2*G*). In the low glucose condition, we observed an elevated level of glutathionylation in PP2Cα WT (Fig. 2*H*, top, lanes 3 vs. 1 and 2) and also in C204S and C238S mutants with similar intensities (Fig. 2*H*, top, lanes 3, 5, and 7). In contrast, an insignificant level of glutathionylation was found in PP2Cα C314S (Fig. 2*H*, top, lanes 8 and 9 vs. 3, and Fig. S7*E*, left). A similar pattern was also observed in MDA-MB-231 cells in response to H_2_O_2_ (0 vs. 1 mM) (Fig. 2*H*, bottom, and Fig. S7*E*, right), supporting that PP2Cα glutathionylation occurs selectively at C314 in cells, which is in agreement with our previous report (43). The observed glutathionylation selectivity at C314 may be attributed to the potential inaccessibility of C204 that results from PP2Cα′s protein–protein interaction or altered conformation in cells (26). Taken together, our data support that PP2Cα glutathionylation occurs selectively at C314 in response to H_2_O_2_ or altered glucose concentrations.

### PP2Cα C314 glutathionylation increases cell migration and invasion

**Figure 3:**
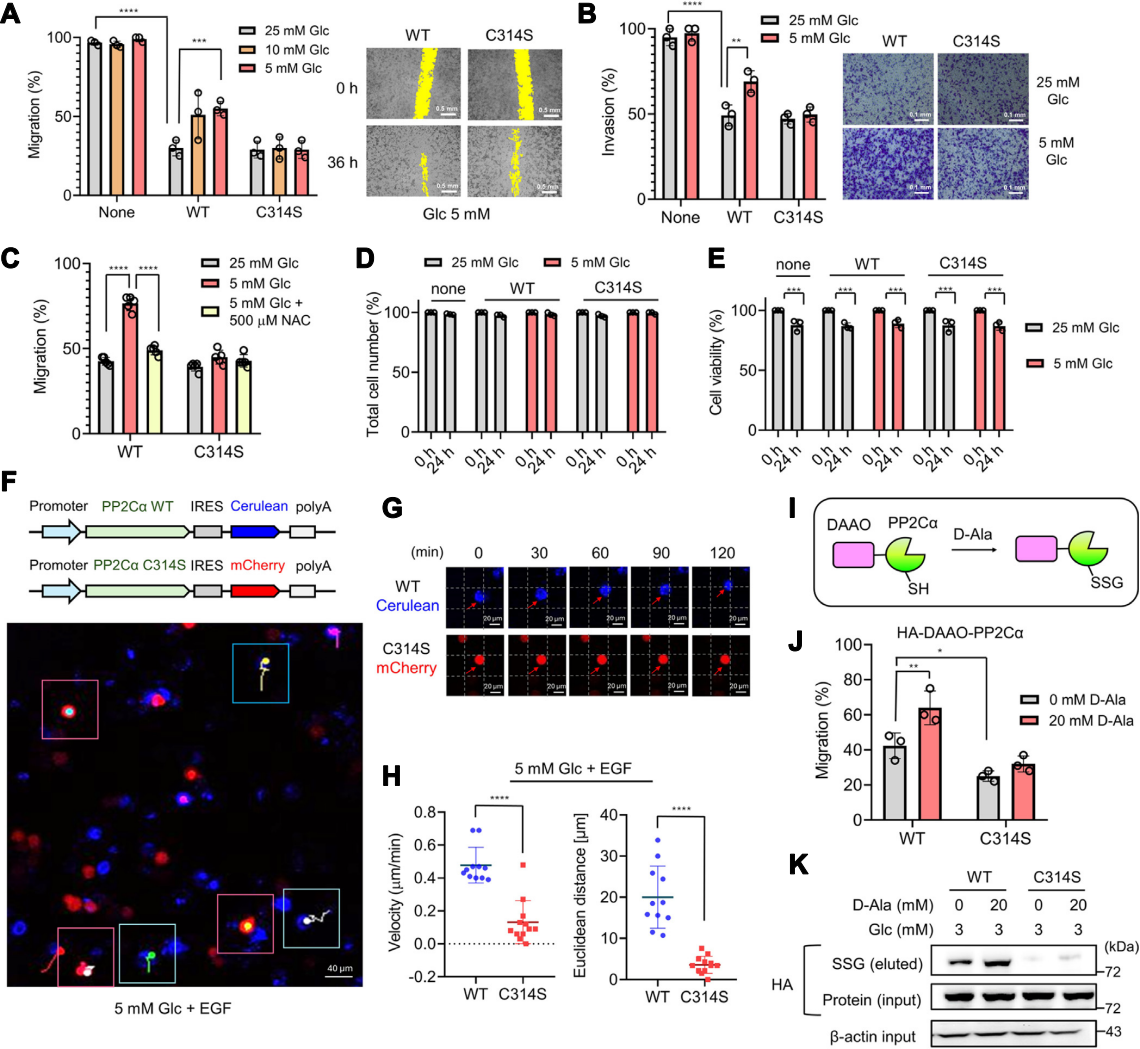
**PP2Cα C314 glutathionylation increases cell migration and invasion.** A–*E*, analyses of MDA-MB-231 cells expressing PP2Cα WT or C314S for migration, invasion, and viability. MDA-MB-231 cells expressing PP2Cα WT or C314S were incubated in different glucose concentrations. *A*, the wound-healing migration assay for 36 h (n = 3). The *yellow* color indicates the area without cells. The scale bar represents 0.5 mm. *B*, transwell-invasion assay for 24 h (n = 3). The scale bar represents 0.1 mm. *C*, the wound-healing migration assay upon adding N-acetylcysteine (NAC) for 24 h (n = 3). *D*, total cell numbers after 24 h (n = 3). *E*, cell viability after 24 h (n = 3). *F*–*H*, tracking migration of individual cells. MDA-MB-231 cells expressing PP2Cα WT/Cerulean (*blue*) or PP2Cα C314S/mCherry (*red*) were combined and incubated in low glucose (5 mM) with EGF for 2 h. Cells were monitored by fluorescence every 10 min (0–120 min). The images were combined to analyze the migration of individual cells (n = 10–12). *F*, bicistronic plasmid maps (*top*) and the confocal image with migration tracks of individual cells (*bottom*). Cells visible during the entire time frame were analyzed. *Red* boxes show *red* cells, whereas *blue* boxes show *blue* cells. In each box, migration tracks over 2 h are shown by tails. The scale bar represents 40 μm. *G*, representative images of individual cells at different time points, showing the migration. The scale bar represents 20 μm. *H*, migration velocity and distance. Individual cells were analyzed and shown in a dot plot. *I*, the PROX-D system. DAAO is fused to the protein of interest (POI) (*i.e.*, PP2Cα), and D-Ala incubation produces H_2_O_2_ in proximity to POI, which causes POI oxidations. *JandK*, cell migration and PP2Cα glutathionylation induced by PROX-D. DAAO-PP2Cα WT or C314S was expressed in MDA-MB-231 cells. Cell migration (n = 3) (*J*) and PP2Cα glutathionylation (n = 3) (*K*) were measured after adding D-Ala to cells in 3 mM glucose for 24 h. Data represent the mean ± SD. The statistical difference was analyzed by two-way ANOVA and Tukey’s *post hoc* test (*A–E*, and *J*) or two-tailed Student’s unpaired *t* test (*H*), where ∗*p* < 0.03, ∗∗*p* < 0.002, ∗∗∗*p* < 0.0002, ∗∗∗∗*p* < 0.0001.

Next, we sought to further investigate cell migration increased by PP2Cα C314 glutathionylation. MDA-MB-231 cells without or with expressing PP2Cα WT or C314S (2.3-fold ectopic expression *versus* endogenous one, Fig. S9*A*) were compared in the wound-healing assay with different glucose concentrations (25, 10, and 5 mM) (Figs. 3*A* and S9*B*). MDA-MB-231 cells without PP2Cα expression showed high migration at all glucose concentrations (Fig. 3*A*, bars 1–3, and S9B, left). Expectedly, PP2Cα overexpression decreased MDA-MB-231 migration (Fig. 3*A*, bar 1 vs. 4) in agreement with PP2Cα′s inhibitory role in proliferation and migration (33). However, cells expressing PP2Cα WT showed a glucose concentration-dependent increase in cell migration (Fig. 3*A*, bars 4–6, and S9*B*, middle). In contrast, cells expressing PP2Cα C314S showed no significant change in cell migration in identical conditions (Fig. 3*A*, bars 7–9, and S9*B*, right), supporting increased cell migration upon PP2Cα C314 glutathionylation or oxidation. Notably, the increased migration in cells with PP2Cα WT induced by low glucose was not seen upon the addition of N-acetylcysteine (NAC) (Figs. 3*C* and S9*C*), a precursor for glutathione synthesis for redox homeostasis, suggesting the redox-dependent increase of cell migration. PP2Cα is a negative regulator of cell proliferation, migration, and invasion (33). Therefore, cell number or proliferation was examined under identical conditions. MDA-MB-231 cells without or with expression of PP2Cα WT or C314 did not show a significant difference in the total number of cells (Fig. 3*D*) while displaying a slight decrease of cell viability in both high and low glucose concentrations (Fig. 3*E*), supporting that PP2Cα C314 glutathionylation does not contribute to cell proliferation in low glucose conditions. However, transwell invasion assay showed increased invasion of PP2Cα WT cells in low glucose compared to high glucose conditions, as opposed to insignificant change of invasion with PP2Cα C314S (Figs. 3*B* and S9*D*), supporting that PP2Cα C314 glutathionylation increases both migration and invasion.

To corroborate the increased migration, we sought to monitor and track cell migration in fluorescence microscopy. To do so, we produced two bicistronic plasmids that enable the expression of two genes with internal ribosome entry site (IRES) element: PP2Cα WT with Cerulean (WT/Cerulean) and PP2Cα C314S with mCherry (C314S/mCherry) (Figs. 3*F* and S10*A*). After transfection of individual plasmids, a similar number of MDA-MB-231 cells with WT/Cerulean or C314S/mCherry were combined and monitored for migration by fluorescence (Fig. S10*A*). A mixture of WT/Cerulean and C314S/mCherry cells was incubated with low glucose (5 mM), along with EGF to enhance cell migration velocity. Notably, WT/Cerulean cells (blue, Fig. 3, *F* and *G* and S10, *B*–C) migrated faster with velocity of 0.48 μm/min (Fig. 3*H,* left) and migration distance of 20.0 μm for 2 h (Fig. 3*H*, right), whereas C314S/mCherry cells (red, Fig. 3, *F* and *G* and S10, *B*–C) migrated slower with velocity of 0.14 μm/min (Fig. 3*H*, left) and migration distance of 3.6 μm for 2 h (Fig. 3*H*, right) under the identical condition (5 mM glucose). The chemotaxis analysis also showed stochastic migration directions but longer tracks of individual cells with WT *versus* C314S (Fig. S10*D*). Taken together, our data support that PP2Cα C314 glutathionylation in oxidative stress induced by low glucose increases the migration of MDA-MB-231 cells.

In addition to the low glucose condition, we sought to demonstrate that PP2Cα glutathionylation induced directly by H_2_O_2_ can increase cell migration. To do so, we developed a system, namely proximity-induced oxidation of a protein by DAAO (PROX-D) (Fig. 3*I*). In this system, a protein of interest (POI) (*i.e.*, PP2Cα WT) fused to DAAO is expressed in cells. D-Ala incubation produces H_2_O_2_ nearby to the POI, thus causing the POI’s oxidation (Fig. 3*I*). The approach may not induce selective oxidation of POI in the proteome. However, it simulates redox signaling where an ROS source or H_2_O_2_ is localized to the POI (47, 51). The wound-healing migration assay confirmed enhanced migration of cells expressing DAAO-PP2Cα WT *versus* C314S upon the addition of D-Ala (Figs. 3*J* and S11*A*). The clickable glutathione approach confirmed increased glutathionylation of DAAO-PP2Cα WT upon adding D-Ala (20 mM), as opposed to DAAO-PP2Cα C314S (Figs. 3*K* and S11, *B–C*), demonstrating that H_2_O_2_-induced PP2Cα glutathionylation at C314 causes elevated migration of MDA-MB-231 cells.

### PP2Cα C314 glutathionylation activates JNK and ERK pathways by selectively altering protein–protein interactions

Next, we sought to analyze signaling pathways or molecular mechanisms of how PP2Cα glutathionylation increases cell migration and invasion. PP2Cα is a negative regulator of MAPK (34), TGFβ/SMAD (36), and NF-kB signaling (37) that are associated with cell migration and invasion. Therefore, we hypothesized that PP2Cα C314 glutathionylation may inhibit its phosphatase activity or protein–protein interactions, such that PP2Cα′s negative role is compromised and its downstream signaling pathways are activated to increase cell migration.

First, PP2Cα phosphatase activity was measured without and with glutathionylation: Purified PP2Cα WT or C314S was glutathionylated by incubating with oxidized glutathione (GSSG) for 2 h, and their phosphatase activities were measured using a p-nitrophenyl phosphate (pNPP) substrate. The kinetic parameters (K_m_ and k_cat_) of PP2Cα WT were essentially the same with and without incubation with GSSG (the k_cat_/K_m_ values of PP2Cα WT: 58 and 55 M^-1^s^-1^ without and with GSSG) (Table 1 and Fig. S12). The same result was seen even with PP2Cα C314S (the k_cat_/K_m_ values of PP2Cα C314S: 48 and 47 M^-1^s^-1^ without and with GSSG) (Table 1 and Fig. S12), supporting that PP2Cα glutathionylation does not change its phosphatase activity, which is in agreement with the distant location of C314 from the active site (Fig. 2*A*).

**Table 1:** PP2Cα enzyme activity without and with glutathionylation

PP2Cα	K_m_ (mM)	k_cat_ (min^−1^)	k_cat_/K_m_ (M^−1^S^−1^)
WT	1.51 ± 0.4	5.22 ± 0.4	57.6
WT + GSSG	1.51 ± 0.5	4.95 ± 0.5	54.6
C314S	1.44 ± 0.2	4.14 ± 0.2	47.9
C314S + GSSG	1.42 ± 0.2	4.03 ± 0.2	47.3

**Figure 4:**
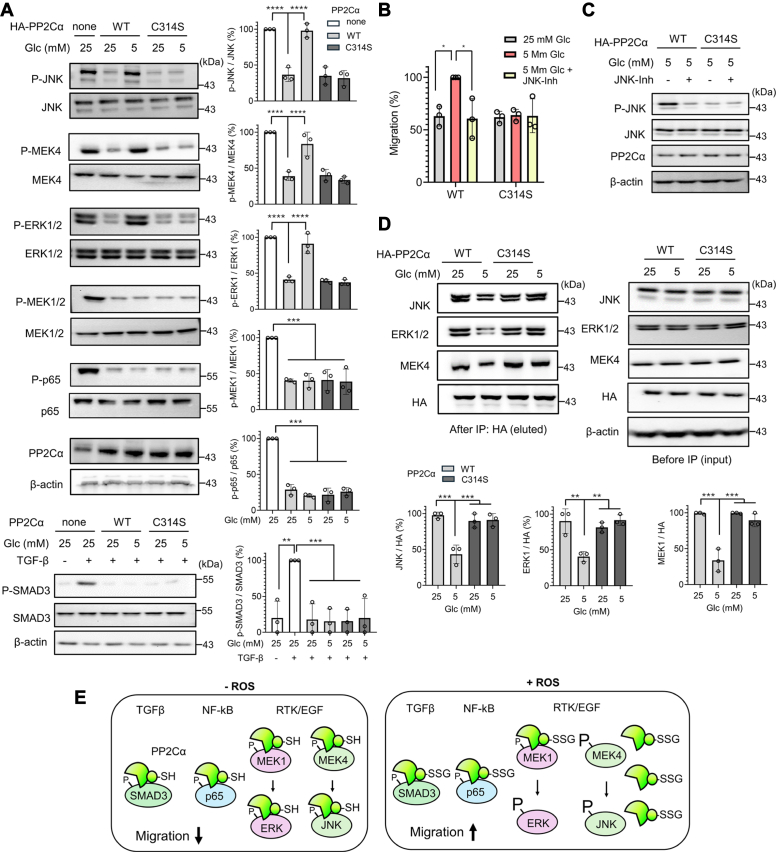
**PP2Cα C314 glutathionylation activates JNK and ERK pathways by selectively altering protein–protein interactions.***A*, activation of JNK, ERK1/2, and MEK4 upon PP2Cα glutathionylation. MDA-MB-231 cells expressing PP2Cα WT or C314S were incubated in low or high glucose for 6 h, and phosphorylation levels of PP2Cα substrates were analyzed by western blots (n = 3). *B*–*C*, JNK activation is responsible for increased cell migration. The wound-healing migration for 24 h (n = 3) (*B*) and JNK phosphorylation after 6 h (n = 3) (*C*) in cells expressing PP2Cα WT or C314S upon the addition of JNK-inhibitor (JNK-Inh). *D*, PP2Cα glutathionylation induces its dissociation from JNK, ERK1/2, and MEK4. After incubation of cells in low or high glucose for 6 h, co-immunoprecipitation was used to monitor their binding interactions by western blots (n = 3). *E*, a model for cell migration induced by PP2Cα glutathionylation. In a nonstressed condition, PP2Cα binds to its substrates for their dephosphorylation, which suppresses cell migration. However, H_2_O_2_ or ROS induced by low glucose causes PP2Cα glutathionylation at C314, which dissociates PP2Cα from selected signaling substrates, such as JNK, ERK1/2, and MEK4. The dissociation increases phosphorylation levels of JNK, ERK1/2, and MEK4, which causes increased cell migration *via* the JNK-paxillin pathway. Data show the mean ± SD or representative of three independent experiments. The statistical difference was analyzed by one-way ANOVA with Tukey’s *post hoc* test (*A*, *D*) or two-way ANOVA with Turkey’s *post hoc* test (*B*), where ∗*p* < 0.03, ∗∗*p* < 0.002, ∗∗∗*p* < 0.0002, ∗∗∗∗*p* < 0.0001.

Next, we examined whether PP2Cα′s downstream substrates are activated upon PP2Cα glutathionylation by measuring phosphorylation levels of PP2Cα substrates. Based on literature and interactome analysis of PP2Cα (Fig. S13), we selected signaling substrates of PP2Cα implicated in cell migration, including MAPK (ERK1/2, JNK1/2, p38) (34, 52), JNK and ERK upstream kinases (MEK4 and MEK1/2, respectively) (53), NF-kB/p65 (38), SMAD2/3 (36), and PAK1/2. Therefore, MDA-MB-231 cells expressing PP2Cα WT or C314S were incubated with high (25 mM) or low (5 mM) glucose. Subsequently, phosphorylation levels of individual substrates were examined by Western blot analysis (Fig. 4*A*). Overexpression of PP2Cα WT or C314 reduced phosphorylation levels of substrates (JNK, MEK4, ERK1/2, MEK1/2, p65, SMAD3) (Fig. 4*A*, lanes 1 vs. 2 for all substrates, except lane 2 vs. 3 in SMAD3), confirming that these proteins are downstream substrates of PP2Cα. In contrast, we observed that two kinases (p38 and PAK1/2) did not alter their phosphorylation levels after PP2Cα overexpression (Fig. S14*A*). Notably, phosphorylation levels of three proteins (JNK, ERK1/2, MEK4) were increased in cells with PP2Cα WT in low glucose compared to high glucose conditions (Fig. 4*A*, lane 2 vs. 3). In contrast, their phosphorylation levels remain unchanged in cells with PP2Cα C314S in both low and high glucose conditions (Fig. 4*A*, lanes 4 vs. 5), suggesting that phosphorylation of JNK, ERK1/2, and MEK4 increases upon PP2Cα C314 glutathionylation. On the other hand, other proteins (MEK1/2, p65, and SMAD3), albeit being PP2Cα′s downstream substrates (Fig. 4*A*), did not increase their phosphorylation levels in PP2Cα WT nor C314S in low glucose (Fig. 4*A*), suggesting that selected substrates (JNK, ERK1/2, and MEK4) among PP2Cα substrates are activated upon PP2Cα glutathionylation. Phosphorylation of JNK, along with its upstream kinase MEK4, is known to increase cell migration *via* paxillin, an important adaptor protein in the adhesion complex (54). In agreement, paxillin phosphorylation was increased in cells expressing PP2Cα WT in low glucose compared to high glucose conditions, whereas no change was seen in cells with PP2Cα C314S (Fig. S14*B*). In addition, the increased migration of cells with PP2Cα WT in low glucose was abrogated by incubation of a JNK inhibitor (Figs. 4*B* and S14*C*) at a concentration that does not cause inhibition of other kinases (*i.e.*, p38) nor cells with PP2Cα C314S (Figs. 4*C* and S14*D*), corroborating the important role of JNK in PP2Cα glutathionylation-mediated cell migration.

Because the PP2Cα enzyme activity is not altered by its glutathionylation, the changes in phosphorylation levels of three proteins (JNK, ERK1/2, MEK4) likely occurred due to changes in PP2Cα′s protein–protein interactions upon PP2Cα C314 glutathionylation. The subsequent co-immunoprecipitation experiments confirmed that PP2Cα WT decreased its interactions with JNK, ERK1/2, and MEK4 in low glucose compared to high glucose (Fig. 4*D*, lanes 1 vs. 2). On the other hand, PP2Cα C314S remained its interactions with JNK, ERK1/2, and MEK4 under identical conditions (Fig. 4*D*, lanes 3 vs. 4), supporting that PP2Cα glutathionylation decreases its interactions with these three kinases. Therefore, these data support our model (Fig. 4*E*) that PP2Cα suppress cell migration by dephosphorylating various substrates, including MEK4, JNK, and ERK1/2, in a nonstressed condition. However, in the presence of ROS, glutathionylated PP2Cα loses its interactions with MEK4, JNK, and ERK1/2, resulting in increases in their phosphorylation levels and contributing to increased cell migration and invasion.

### PP2Cα C314 glutathionylation constitutes EGF-mediated redox signaling

**Figure 5:**
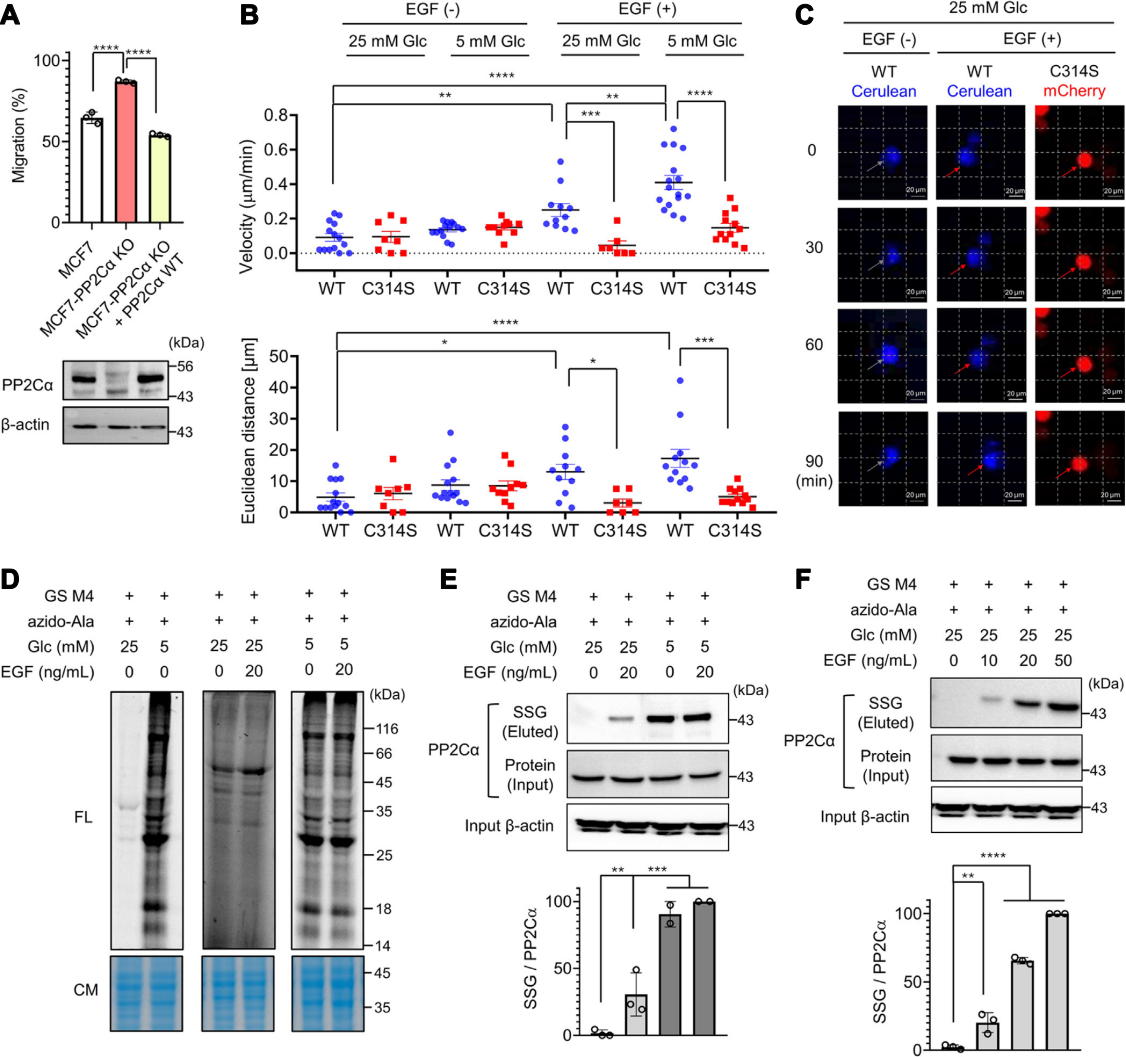
**PP2Cα C314 glutathionylation constitutes growth factor–mediated redox signaling.***A*, migration of MCF7-PP2Cα KO cell lines without and with ectopic expression of PP2Cα. The wound-healing migration of MCF7-derived cell lines in high glucose (25 mM) for 24 h (*top*) and PP2Cα levels in the cell lines (*bottom*) (n = 3). *B*–*C*, migration velocities and distances of individual MCF7-PP2Cα KO cells expressing PP2Cα WT (MCF7-PP2Cα WT) or C314S (MCF7-PP2Cα C314S). MCF7-PP2Cα WT with Cerulean (*blue*) and MCF7-PP2Cα C314S with mCherry (*red*) were combined and incubated in low or high glucose without or with EGF for 1.5 h. *B*, individual cells (n = 7–14) were monitored and analyzed for migration distances and velocities. *C*, representative images of individual cell migration. The scale bar represents 20 μm. *D*–*F*, EGF induces PP2Cα glutathionylation in redox signaling. Global glutathionylation (n = 3) (*D*) and PP2Cα glutathionylation (n = 3) (*E*–*F*) in MCF7 upon adding EGF for 16 h. After incubation of EGF, lysates were click-conjugated with rhodamine-alkyne (*D*) or biotin-alkyne (*E–F*). Glutathionylated proteins were analyzed by fluorescence (FL, SSG levels) with Coomassie stains (CM, protein levels) (*D*) or western blots after (SSG level) and before (protein level) enrichment by streptavidin-agarose (*E–F*). Data show the mean ± SD or representative of three independent experiments. The statistical difference was analyzed by one-way ANOVA and Tukey’s (*A*, *B*, *E*) or Dunnett’s (*F*) *post hoc* test, where ∗*p* < 0.03, ∗∗*p* < 0.002, ∗∗∗*p* < 0.0002, ∗∗∗∗*p* < 0.0001.

To demonstrate the role of PP2Cα glutathionylation beyond MDA-MB-231 cells, we extended our experiment to an epithelial MCF7 cell line that harbors a higher level of endogenous PP2Cα (Fig. S15*A*) (42). We used a CRISPR/Cas9 system to generate MCF7 cell line with PP2Cα knockout (MCF7-PP2Cα KO) and confirmed a complete knockout of endogenous PP2Cα by Western blot (Fig. S15, *B*–*C*). The subsequent wound-healing migration assay showed that MCF7-PP2Cα KO cells migrate faster than parental MCF7 cells (Fig. 5*A*, bars 1 vs. 2 and Fig. S15*D*). In contrast, the enhanced migration of MCF7-PP2Cα KO cells was ablated upon expression of PP2Cα WT to a comparable level to parental MCF7 cells (Fig. 5*A*, bars 1 vs. 3, and Fig. S15*D*), confirming a negative role of PP2Cα in cell migration. Next, PP2Cα WT or C314S was re-expressed in MCF7-PP2Cα KO cells. The subsequent wound-healing assay confirmed that MCF7 PP2Cα KO cells with PP2Cα WT increased cell migration in low glucose compared to high glucose conditions, whereas MCF7 PP2Cα KO cells with PP2Cα C314S did not show increased cell migration (Fig. S15*E*).

A seminal example of redox signaling involves ROS or H_2_O_2_ induced by growth factors that oxidize and inhibit the reactive cysteines in the active sites of PTPs, which enhances the activities of kinases, such as MAPK (19, 55, 56). Because we found that PP2Cα C314 glutathionylation antagonizes PP2Cα′s function and activates MAPK (*i.e.*, JNK and ERK1/2), we envisioned that PP2Cα C314 glutathionylation may constitute growth factor (*e.g.*, EGF)-induced redox signaling that regulates cell migration. Therefore, we sought to examine whether PP2Cα C314 glutathionylation contributes to cell migration induced by a growth factor, such as EGF. To do so, MCF7-PP2Cα KO cells expressing PP2Cα WT/Cerulean or PP2Cα C314/mCherry (MCF7-PP2Cα WT or C314, respectively) were combined to monitor and compare their migration in response to EGF in high or low glucose conditions (Figs. 5*B* and S16, *A* *and* *B*). In a high glucose condition (*i.e.*, nonoxidizing environment), both cohorts of cells with PP2Cα WT (blue) or C314 (red) did not migrate significantly without EGF within 1.5 h (ca. ∼0.1 μm/min and 5–6 μm for 1.5 h) (Fig. 5*B*, columns 1–2 and 5C, column 1). However, the addition of EGF caused higher velocity (0.25 vs. 0.05 μm/min) and distance (13.0 vs. 3.0 μm) of migration for cells with PP2Cα WT *versus* C314 (Fig. 5*B*, columns 5–6, and 5C, column 2–3). These data argue that EGF-induced cell migration is partly attributed to PP2Cα C314 glutathionylation or oxidation. Similarly, in a low glucose condition without the addition of EGF, cells with PP2Cα WT or C314 migrated without a significant difference in migration velocity (0.14–0.15 μm/min) and distance (∼8.5 μm for 1.5 h) (Fig. 5*B*, columns 3–4). The nonsignificant difference in migration between low and high glucose conditions (Fig. 5*B*, columns 1–2 vs. 3–4) is presumably due to a short monitoring time (1.5 h) and slow cell migration without EGF. However, upon the addition of EGF in the presence of low glucose, significantly higher migration velocity (0.41 vs. 0.15 μm/min) and distance (17.3 vs. 5.1 μm) were noted for cells with PP2Cα WT *versus* C314S (Fig. 5*B*, columns 7–8), which is consistent with the data seen in MDA-MB-231 cells (Fig. 3*H*).

Next, we analyzed whether PP2Cα glutathionylation can occur upon the addition of EGF. The clickable glutathione approach was used with MCF7 cells. The incubation of cells with EGF in high glucose did not increase significant signals of global glutathionylation (Fig. 5*D*, lane 3 vs. 4). In contrast, as expected, low glucose induced globally higher levels of glutathionylation (Fig. 5*D*, lane 1 vs. 2). However, the addition of EGF to cells in low glucose did not further increase levels of glutathionylation significantly (Fig. 5*D*, lanes 5 vs. 6). Despite a lack of global change of glutathionylation, the subsequent click reaction and pull-down experiments demonstrated that PP2Cα formed glutathionylation upon the addition of EGF in high glucose (Fig. 5*E*, lane 1 vs. 2). PP2Cα glutathionylation was highly enhanced in low glucose compared to high glucose conditions (Fig. 5*E*, lane 3 vs. 1), while the addition of EGF caused a limited increase of glutathionylation in low glucose (Fig. 5*E*, lane 3 vs. 4). Notably, increasing concentrations of EGF induced higher PP2Cα glutathionylation (Fig. 5*F*). EGF induced a high level of glutathionylation in PP2Cα WT but not significantly in PP2Cα C314S (Fig. S17). Taken together, our data support that PP2Cα C314 forms glutathionylation in response to EGF, contributing to the increased cell migration.

## Discussion

A large amount of evidence has demonstrated that ROS play an essential role in cell migration, adhesion, and EMT, where the activities of many signaling proteins and enzymes are directly or indirectly altered by ROS (5, 17). It is well known that ROS-induced protein cysteine oxidations serve as a molecular mechanism for regulating cellular processes (26). Accordingly, evidence continues to illustrate examples of various protein cysteine oxidations, including glutathionylation, regulating cell migration, and adhesion. For example, glutathionylation of MKP-1 under metabolic stress enhances the adhesion and migration of monocytes (27). Metabolic stress also induces glutathionylation of 14-3-3 zeta, a negative regulator of slingshot phosphatase (SSH1L), contributing to monocyte migration (28). Actin glutathionylation was shown to stimulate chemotaxis-mediated migration and adhesion of neutrophils (30). LMW-PTP glutathionylation was found to activate focal adhesion kinase and enhance the migration of endothelial cells (29). In addition to glutathionylation, Src cysteine sulfenylation activates its kinase activity (57), which is associated with cell migration. Integrin (intramolecular) disulfide formation occurs in response to H_2_O_2_, enhancing vascular cell adhesion (58). Cofilin cysteine oxidation occurs during cell polarization and motility, altering actin dynamics (6). Sulfonic acid formation of 15-PDGH promotes its degradation, contributing to the EMT process (59). In addition to Cys, Met oxidation of actin by Mical modulates actin dynamics and controls cell migration and adhesion (60, 61). However, despite these increasing examples, it remains challenging to identify the specific proteins and cysteines regulating cell motility.

In this report, we discovered new glutathionylated cysteines regulating cell migration. Our approach involved integrating an existing glutathionylation database and bioinformatics to select a list of candidate cysteines (74 Cys in 37 proteins) involved in migration and susceptible to glutathionylation. The subsequent biological screening of such candidate cysteines (14 Cys in nine proteins) in migration assays led to the identification of three new proteins and their cysteines (PP2Cα C314, ARHGEF7 C490/C721, NISCH C185) that regulate cell migration *via* their glutathionylation or oxidation. The significance of our approach is that it can be extended to other biological models, such as inflammation and apoptosis, streamlining the identification of “regulatory cysteines” linked to a specific “biological process.” It is also envisioned that investigating remaining cysteines in the list of candidates (74 Cys in 37 proteins) may identify new regulatory cysteines for cell migration.

In this report, out of three proteins identified, we demonstrated that PP2Cα C314 glutathionylation increases cell migration and invasion. It is interesting to note that C314 is positioned at CTD, a small domain not fully known for its function to our knowledge, although it was suspected that CTD might contribute to PP2Cα substrate specificity (62). Our data demonstrate that PP2Cα CTD is important for binding interactions with its substrates, including JNK, ERK1/2, and MEK4, as glutathionylation at C314 in CTD caused PP2Cα′s dissociation from these three proteins. It is also interesting to find that PP2Cα C314 is not conserved among its mammalian orthologs nor with its paralog PP2Cβ (PPM1B), which has high sequence similarity (75%) and overlapping functional substrates, suggesting that the regulatory function of PP2Cα C314 likely has evolved at a late evolutionary stage. This idea is consistent with recent findings that redox regulatory cysteines are found in less conserved cysteines or only conserved in evolutionarily related organisms (24, 63, 64). Moreover, it is worth discussing that although both C204 and C314 have higher oxidation susceptibility features (*i.e.*, low pK_a_ and high ASA) and were identified for their glutathionylation *in vitro*, C314 was selectively glutathionylated in the cell environment. The finding suggests that other factors, beyond pK_a_ and ASA, significantly contribute to glutathionylation susceptibility in cells (31), which is consistent with recent findings that protein conformation (or protein–protein interaction) could serve as a determining factor for glutathionylation susceptibility (26).

Cysteine oxidation by H_2_O_2_ involves sulfenylation, sulfinic and sulfonic acid formation, and disulfide bond formation in addition to glutathionylation. In this report, we developed a new strategy to support that H_2_O_2_-induced PP2Cα C314 oxidation or glutathionylation is responsible for the observed phenotype (*i.e.*, cell migration). Specifically, we developed a system, PROX-D, in which DAAO is fused to PP2Cα, thus increasing H_2_O_2_ production near PP2Cα. We envisioned that PP2Cα-DAAO fusion may preferentially oxidize PP2Cα cysteine(s) along with other nearby proteins, especially by simulating redox signaling where an oxidase is in proximity to target proteins. The PROX-D approach evidently supported that DAAO/H_2_O_2_-mediated glutathionylation on PP2Cα is responsible for the observed cell migration. As the approach is modular, it would be applicable to other proteins of interest in future studies. However, it is important to point out that although we have not detected other cysteine oxidations than glutathionylation, our studies do not exclude a possibility of other cysteine oxoforms for conferring the same phenotype as glutathionylation. Additional experiments will be necessary to understand the roles of other cysteine oxidations on PP2Cα C314 in future studies.

PP2Cα is involved in multiple signaling pathways. This report focused on evaluating phosphorylation levels of selected PP2Cα substrates. We found that JNK, ERK1/2, and MEK4 phosphorylation mediates the effect of PP2Cα glutathionylation, whereas phosphorylation of NF-kB (p65) and TGF-β/SMAD3 is not altered. In addition to MAPK and TGF-β/SMAD3 pathways, PP2Cα dephosphorylates other substrates (35). Recent findings demonstrated that PP2Cα is abundantly expressed in the immune cells (*e.g.*, macrophage and monocytes) where it plays a key role in negating anti-viral and anti-bacterial defense of the innate immunity by dephosphorylating various substrates, including JNK, MAVS, TBK1/IKKβ, and p62-SQSTM1 (40, 65). Therefore, it will be interesting to investigate whether PP2Cα glutathionylation modulates these other substrates in the immune cells.

Furthermore, it is worth discussing that PTPs (*e.g.*, PTP1B, SHP1/2, and PTEN) are typically thought to be susceptible to oxidation in growth factor–mediated redox signaling because of their conserved reactive cysteines in the active sites (66). Indeed, two phosphatases (MKP-1 and LMW-PTP) regulating cell migration *via* glutathionylation belong to a family of PTP or dual phosphatase with reactive cysteines (C258 and C13, respectively) in the active site (27, 29). In contrast, Ser/Thr phosphatases do not harbor such reactive cysteines, thus not primarily considered for oxidations in redox signaling. Therefore, evidence for Ser/Thr phosphatases to be a part of redox signaling remains scarce, although the majority of protein phosphorylation is estimated on Ser/Thr in eukaryotic cells (67). Previously, Ser/Thr phosphatase PP2A has been noted for its oxidation susceptibility (68). However, its specific cysteine(s) for oxidation is less clarified. PP2B calcineurin is also known for its regulation by oxidants but likely *via* Fe^2+^/Zn^2+^ oxidation in the active site *versus* cysteine oxidation (69). In this report, we provide evidence that EGF stimulus can increase glutathionylation of PP2Cα, a prototype class PPM Ser/Thr phosphatase, contributing to enhanced cell migration of MCF7. Therefore, our report provides an example of Ser/Thr phosphatase in growth factor redox signaling, supporting that a Ser/Thr phosphatase, in addition to PTPs, participates in growth factor signal transduction.

Although we focused on analyzing PP2Cα C314 glutathionylation in this report, our data support glutathionylation of two other proteins, ARHGEF7 and NISCH, with their roles in regulating cell migration. ARHGEF7 (beta-Pix) is one of the Rho GTPase exchange factors involved in activating Rac1 and Cdc42, which are key regulators of cell migration *via* cytoskeletal reorganization (70). Many reports support the critical role of ARHGEF7 in cell spreading and motility associated with wound healing, axon formation, and cancers (71, 72, 73, 74). ARHGEF7 upregulation is observed in metastatic cancers that increase migration and invasion (73). Therefore, our data lead us to hypothesize that ARHGEF7 C490/C721 glutathionylation may positively activate its function, thus increasing cell migration. NISCH (Nischarin) is a protein that interacts with a cytosolic tail of α5-integrin and negatively regulates integrin signaling related to cell migration (75). NISCH is ubiquitously expressed in the mammary gland and frequently expressed at low levels in breast cancers (76). There is a strong correlation between NISCH-positive cancer patients and increased overall survival (76). Overexpression of NISCH has shown inhibition of migration and invasion of breast cancer cell lines *via* inhibition of Rac1, Cdc42, and PAK1 (75). Therefore, NISCH glutathionylation may antagonize its function, increasing cell migration. Future studies will be necessary to demonstrate and characterize glutathionylation of ARHGEF7 and NISCH.

Lastly, it is worth discussing that we observed increased cell migration in response to low glucose (5 mM and 1 mM) compared to high glucose (25 mM) concentrations. Typically, 5 mM is considered a normal physiological glucose concentration in plasma, whereas 1 mM and 25 mM glucose concentrations are hypoglycemic and diabetic conditions. Many reports demonstrated that 25 mM glucose induces ROS elevation or oxidative stress (77, 78, 79, 80, 81), which contributes to diabetic symptoms. On the other hand, several reports suggest seemingly contradictory results, where high glucose (25 mM) reduces ROS levels and helps maintain redox homeostasis (82, 83, 84), likely *via* its use to form NADPH, an essential reducing equivalent in cells. The discrepancy may be partly attributed to different cell lines and experimental conditions. For example, cancer cell lines in our studies were maintained in Dulbecco’s modified Eagle’s medium (DMEM), which contains high glucose (25 mM). These cancer cell lines may be adjusted to or require the high glucose (25 mM) concentration to maintain redox homeostasis. Therefore, in our studies, glucose concentrations lower than 25 mM (*i.e.*, one or 5 mM) may be insufficient to maintain a redox balance, causing ROS elevation and global glutathionylation (Fig. 2*G*). Our observation implies that the compromised glucose concentration (*e.g.*, either hypoglycemia or cancer cells experiencing insufficient glucose availability) can alter a redox state, increasing ROS, glutathionylation, and cell migration.

## Experimental procedures

### Bioinformatics

Glutathionylated proteins were identified in mouse HL-1 cell line in the previous publications (44, 48, 49), and PP2Cα glutathionylation was found in human embryonic kidney 293 (HEK293) cell line, which were combined as a list of glutathionylated proteins (Table S1). For STRING analysis using Cytoscape software, mouse proteins identified for glutathionylation (n = 1373) were converted to human orthologs (n = 1351) and loaded into the STRING program as a “glutathionylation” network. A list of migration-related proteins was retrieved from the STRING database to produce a “migration” network. Two networks were merged by Cytoscape to identify proteins that belong to both networks. This merged network was subjected to cluster analysis with 50 interacting proteins by MCL clustering in Cytoscape with a granularity parameter 4 and array sources from a score. The merged network was submitted to DAVID GO analysis (https://david.ncifcrf.gov/) to analyze their biological processes.

### Phosphatase enzyme assay

PP2Cα WT or C314S mutant was incubated with and without 0.5 mM oxidized glutathione (GSSG) in 20 mM Tris, pH 7.4 for 2 h at 37 °C. The buffer was replaced by 20 mM Tris HCl, pH 7.4, using Amicon ultra centrifugal filters (Millipore Sigma, Cat# UFC503024). Phosphatase assay was performed with 5 μg PP2Cα in the assay buffer containing 20 mM Tris, pH 7.4, 5 mM MgCl_2_, 0.1 mg/ml bovine serum albumin (BSA), and varying amounts of pNPP (0–20 mM). The absorbance was measured at 405 nm in a kinetic mode for 10 min using the UV-Vis spectrophotometer (Beckman). The absorbance (Ab) was plotted against time for each pNPP concentration, and the slope was calculated (Ab/min). The rate was converted to μM/min using the Beer-Lambert law, where the molar extinction coefficient of pNPP (18,000 M^-1^ cm^-1^) and the 1 cm path length were used. The rates and concentrations of pNPP were used in nonlinear regression in GraphPad Prism software to analyze the Michaelis–Menten kinetic parameters.

### Glutathionylation analysis by in-gel fluorescence

To induce glutathionylation *in vitro*, purified PP2Cα (10 μg) was incubated with 1 mM azido-glutathione (N_3_-GSH) and different concentrations (0–200 mM) of H_2_O_2_ in 1× PBS (pH 7.4) for 15 min or 1 mM oxidized azido-glutathione (N_3_-GSSG-N_3_) in 1× PBS (pH 8) for 30 min. Glutathionylation was quenched by adding iodoacetamide (20 mM) for 20 min at 37  °C in the presence of 1% SDS. Alternatively, glutathionylation in cells was induced, as described in glutathionylation analysis in cells, and cell lysates were prepared.

After inducing glutathionylation, proteins in cell lysates (100 μg) or purified proteins (10 μg) were precipitated by incubating with ice-cold acetone (4× volume) for 1 h at −20 ^°^C and centrifugation for 5 min at 6000 rpm. The supernatant was removed. The pellet was air-dried for 5 min and resuspended in 40 μl buffer containing 0.1% SDS, 1× PBS, and water. The pellet was resuspended entirely by sonication. Cy5-alkyne or rhodamine-alkyne (0.5 μl, 10 mM in DMSO) was added, followed by the click solution (10 μl) containing 20 mM CuBr in DMSO/tBuOH (3:1, v/v; 5 μl) and 20 mM THPTA (5 μl). The reaction mixture was incubated in the dark for 1 h at room temperature. Proteins were separated by SDS-PAGE, and proteins in the gel were analyzed for Coomassie stain and fluorescence by the iBright imaging system (Thermo Fisher Scientific).

### Mass spectrometry

Purified PP2Cα (200 μg) in PBS was reduced with DTT (0.1 mM) for 15 min at room temperature and incubated with oxidized azido-glutathione (1 mM) for 2 h. The unreacted cysteines were blocked with iodoacetamide (20 mM) in the presence of 1% SDS at 37 °C in the dark for 20 min. The protein was precipitated by adding pre-chilled acetone (4 times the reaction volume) and incubating at −20 °C for 30 min. After centrifugation, the protein pellet was redissolved in a click reaction buffer containing 0.2% SDS, PBS, and water. The click reaction was initiated by adding biotin-DADPS-alkyne (400 μM), followed by THPTA (2 mM) and copper bromide (2 mM). The reaction mixture was incubated at room temperature for 1 h. The protein was precipitated with pre-chilled acetone and pelleted by centrifugation. The protein pellet was redissolved in 1.2% SDS in PBS. The protein suspension was added to pre-washed streptavidin agarose beads (50 μl) in PBS. The solution was incubated overnight at 4 °C and 3 h at room temperature the following day. The supernatant was removed, and the streptavidin beads were washed with 0.2% SDS and PBS. The protein on the beads was denatured with 6 M urea in PBS at 37 °C for 45 min. After denaturation, the protein was digested with 2 μg trypsin in 2 M urea and 1 mM calcium chloride in PBS for 3 h at 37 °C. Additional trypsin (2 μg) was added, and the digestion continued overnight. On the following day, the trypsin digestion mixture was removed, and the beads were washed with 0.2% SDS, PBS, and distilled water. For acidic cleavage of the modified peptides, 10% formic acid (50 μl) was added to the beads and incubated at room temperature for 30 min three times. The eluted peptides were collected, lyophilized, and subjected to MALDI-TOF analysis.

The eluted sample was also analyzed by LC-MS/MS, as described previously (31, 85). The sample was run in an LC-MS/MS system consisting of an Easy-nLC 1200 (with an in-house packed C18 nano-column) coupled to a Fusion Orbitrap (Thermo Fisher Scientific). MS spectrum for parent ions (with MS1 scan from m/z 400–1800) was collected in orbitrap with a scan cycle of 3 s at a resolution of 120,000. Parent ions in a charge state of +2 to 6 with an intensity higher than 5000 were analyzed by HCD-induced fragmentation (MS2). MS2 spectra were detected at a resolution of 30,000. The raw data file was generated by the instrument. An ms2 file containing MS2 spectra was extracted using RawConverter (version 1.1.0.23) (publicly available at http://fields.scripps.edu/rawconv). The parent mass (MS1) was searched within the Integrated Proteomics Pipeline (IP2) software with the following modifications on cysteine: azido-glutathione modification - C_16_H_24_N_6_O_7_S; 444.14272.

### Cell culture

HEK293 cell line stably expressing GS M4 (HEK293-GS M4) (43), breast cancer cell line MCF7 (derived from a 69-year-old white female) (ATCC, HTB-22), and breast cancer cell line MDA-MB-231 (derived from a 51-year-old white female) (ATCC, HTB-26) were maintained in DMEM supplemented with 10% fetal bovine serum (FBS, Hyclone, Cytiva), penicillin (100 units/ml), and streptomycin (100 μg/ml) (Pen-Strep, Invitrogen) at 37 °C in a 5% CO_2_ environment.

### Transfection

Lipofectamine-3000 (Thermo Fisher Scientific, Cat# L3000015) transfection was done when cells were 80% confluent. The following amounts of DNA and reagents were used for transfection to HEK293/GS M4 cells in a 10 cm dish. In an Eppendorf tube, 8 μg plasmid DNA and 16 μl P3000 (DNA: P3000 = 1:2) were mixed in 500 μl Opti-MEM medium. In a separate tube, 24 μl Lipofectamine-3000 reagent was mixed with 500 μl Opti-MEM medium. Two tubes were mixed into one tube (1 ml total) and incubated for 10 min at room temperature. DMEM in the dish was replaced by 9 ml Opti-MEM, and a DNA-lipofectamine mixture was added to the dish (10 cm). The solution was mixed by rocking the dish and incubated in a humidified incubator at 37 °C and 5% CO_2_ for 5 to 6 h. Opti-MEM was then replaced by DMEM medium supplemented with 10% FBS and 1% Pen-Strep, and cells were returned to the humidified incubator at 37 °C and 5% CO_2_. For MDA-MB-231, MCF7, and MCF7-PP2Cα KO cell lines, 14 μg DNA, 28 μl P3000, and 21 μl Lipofectamine-3000 were used for a 10 cm dish.

### Glutathionylation analysis in cells

HEK 293/GS M4 cells or MDA-MB-231 cells were transfected with HA-PP2Cα WT or C314S plasmid. The next day, GS M4 was expressed in MDA-MB-231 cells by infecting adenovirus carrying GS M4 (Ad-GS M4). Briefly, 7.5 μl of adenovirus (9 × 10^10^ PFU/ml) and 8 μl of 10 mg/ml polybrene were mixed in 500 μl DMEM supplemented with 2% FBS. Cells were incubated with the adenovirus solution for 6 h and subsequently in DMEM with 10% FBS for 18 h. After expression of GS M4, cells were incubated with 0.6 mM azido-Ala for 20 h to synthesize clickable azido-glutathione. Cells were then treated with 1 mM H_2_O_2_ for 15 min (HEK293/GS M4 cells) to induce glutathionylation. Alternatively, cells were incubated with 5 or 25 mM glucose in glucose-free DMEM for 6 h. For DAAO-mediated glutathionylation of PP2Cα, cells were transfected with DAAO-PP2Cα constructs, infected with Ad-GS M4, and incubated with 0.6 mM azido-Ala for 20 h. Cells were then incubated with 0 mM or 20 mM D-Ala in 3 mM glucose for 12 h. To analyze PP2Cα glutathionylation upon EGF treatment, MCF-7 cells without or with transfected PP2Cα WT or C314S were infected with Ad-GS M4 and incubated with 0.6 mM azido-Ala for 20 h. Cells were then incubated with 25 or 5 mM glucose without or with 0 to 50 ng/ml EGF for 16 h.

After induction of glutathionylation, cells were lysed using the RIPA lysis buffer containing 1% NP-40, 0.25% sodium deoxycholate, 150 mM NaCl, 1 mM EDTA, 50 mM Tris (pH 8.0), a protease inhibitor cocktail, and 50 mM N-ethylmaleimide. One milligram of lysate was precipitated by adding ice-cold acetone (4× volume) and incubated at −20 °C for 30 min. After centrifugation at 13,000 rpm for 3 min, the supernatant was removed, and proteins were resuspended in a resuspension buffer (40 μl) containing 10× PBS (pH 7.4, 5 μl), 10× SDS (1 μl), and water. The pellet was completely dissolved by sonication. To the solution were added 5 mM biotin-alkyne (4 μl) and the click solution (10 μl), pre-prepared by mixing 20 mM THPTA (5 μl) and 20 mM Cu(I)Br dissolved in DMSO/tBuOH (3:1, v/v; 5 μl). The mixture was incubated for 1 h at room temperature. After the click reaction, proteins were precipitated by incubation with ice-cold acetone (4× volume) for 1 h at −20 °C and centrifugation at 13,300 g for 5 min. The resulting pellet was resuspended in PBS buffer (200 μl) containing 1.2% SDS by sonication. Resuspended proteins (10 μl) were saved for the gel. The remaining proteins were added to PBS buffer (0.2% SDS) containing streptavidin-agarose beads (20 μl, Pierce) and incubated overnight at 4 °C. Beads were washed with PBS containing 0.2% SDS (3 × 500 μl) and PBS (3 × 500 μl). Proteins on beads were eluted by adding 50 μl SDS-loading dye (2×) containing β-mercaptoethanol (3 μl) and heating at 95 °C for 10 min. Eluted proteins were separated by SDS-PAGE and transferred to the polyvinylidene difluoride (PVDF) membrane. The membrane was blocked with 5% BSA in Tris-buffered saline with Tween 20 (TBST) (50 mM Tris–HCl, 150 mM NaCl, and 0.1% Tween-20) and incubated with primary antibody solutions, including hemagglutinin (HA) (1:1000), PP2Cα (1:1000), actin (1:1000), diluted in a blocking buffer overnight at 4 °C. The corresponding horseradish peroxidase (HRP)-conjugated secondary antibodies, anti-mouse IgG (1:2000) and anti-rabbit IgG (1:3000), were used to visualize the proteins by chemiluminescence (SuperSignal West Pico). Blots were analyzed using the iBright imaging system (Thermo Fisher Scientific).

### Migration assay

Nontransfected or transfected cells with WT or C/S mutants were seeded into a 12-well plate to produce a fully confluent monolayer (1.5 × 10^5^ cells). The next day, the cell monolayer was scratched using a 10 μl pipet tip to create an even wound. Cells were washed three times with warm PBS (500 μl × 4) to remove detached cells. Glucose-free DMEM supplemented with the indicated glucose concentration was added to the cells. Alternatively, cells were incubated with different concentrations of H_2_O_2_ (0–100 μM) or the indicated concentrations of D-Ala in DMEM. The wound area was imaged using a light microscope connected to a camera. Cells were then incubated in a humidified incubator at 37 °C and 5% CO_2_ for 24 to 48 h, and the wound area was imaged again. Images were analyzed using an MRI wound healing tool in ImageJ software.

### Transwell invasion assay

After transfection of HA-PP2Cα WT or C314S plasmid, MDA-MB 231 cells (5 × 10^5^ cells) were added to the upper chamber coated with Matrigel. The upper chamber was filled with 200 μl 5 mM or 25 mM glucose in glucose-free DMEM. The lower chamber was filled with 750 μl 10% FBS in glucose-free DMEM. After 24 h, the medium was removed from the lower and upper chambers. Cells were washed three times with PBS, and noninvasive cells were removed using a cotton swab. The bottom side of the transwell insert membrane was fixed with 500 μl 4% formaldehyde for 10 min at room temperature, followed by permeabilization with 500 μl PBST (pH-7.4, 50 mM Tris–HCl, 150 mM NaCl, 0.1% Triton-X100) for another 10 min. Inserts were washed once with PBS and stained with 500 μl 0.2% (W/V) crystal violet solution for 20 min at room temperature. Inserts were then extensively washed with PBS to remove all excess dye. Images were captured using a camera attached to a microscope. The stained dye was extracted with 300 μl 33% acetic acid, and absorbance was measured at 560 nm using a Synergy H1 microplate reader (BioTek).

### Trypan blue viability assay

Cells were trypsinized. Cells were centrifuged at 1000 rpm for 2 min, and the cell pellet was resuspended in 250 μl DMEM. Fifty microliters of cell solution was mixed with 50 μl Trypan blue reagent. Ten to twenty microliters were loaded onto the slide and analyzed by a cell counter (LUNA II, Logos Biosystems).

### Single-cell tracking *via* fluorescence

MDA-MB-231 cells or MCF7-PP2Cα KO cells were transfected with pIRES-PP2Cα WT-Cerulean or pIRES-PP2Cα C314S-mCherry vectors. After 24 h of transfection, cells were sorted using fluorescence-activated cell sorting (FACS). Two cohorts of cells (expressing PP2Cα WT/Cerulean or C314S/mCherry) were combined and seeded together to produce 10 to 20% confluency in a 35 mm dish with glass at the bottom (MatTek, Cat# P35G-1.5-14-C). Cells were incubated overnight at 37 °C with 5% CO_2_. The next day, cells were incubated with 25 mM or 5 mM glucose in glucose-free DMEM and 0 or 20 ng/ml EGF. Cerulean and mCherry were excited at 435 nm and 587 nm and emitted at 477 nm and 610 nm, respectively. Fluorescence images were captured every 10 min for 2 h. Manual tracking and chemotaxis tools in ImageJ were used to analyze the images. Briefly, after uploading images to ImageJ, the manual tracking option under plugins was selected. Time interval and x/y calibration values of the images were set under parameters, and the movement of each cell was manually tracked using the option “add track.” When the tracking was completed, the option “end track” was selected. The distance and velocity information were obtained by selecting “overlay dots and lines” and saving the file as .txt. All cells visible during the entire time frame were selected and analyzed, but those cells appearing or disappearing (out of focus) in the middle of a time frame were not analyzed. Chemotaxis plots were obtained by importing the .txt file in the chemotaxis tool under ImageJ plugins. Before importing the data set, the time interval and the x/y calibration were entered in the setting section. After importing the file, the option “plot graph” was selected under the plot feature. The corresponding trajectory plots and velocity/distance statistics were obtained under the “diagram feature” and “statistic feature” tabs.

### Western blot

To analyze phosphorylation levels, MDA-MB 231 cells transfected with PP2Cα WT or C314S were incubated in 25 mM or 5 mM glucose for 5 h. For the analysis of phosphorylated SMAD2/SMAD3, cells were incubated in 25 mM or 5 mM glucose with 6 ng/ml TGF-β for 4 h. Cells were then lysed with 200 μl RIPA buffer (1% NP-40, 0.25% sodium deoxycholate, 150 mM NaCl, 1 mM EDTA, 50 mM Tris, pH 8.0) in the presence of PhosSTOP phosphatase inhibitor (Sigma Millipore, Cat# 4906845001) and protease inhibitor cocktail (Thermo Fisher Scientific, Cat# A32955). Proteins (40 μg) were resolved by SDS-PAGE and transferred to the PVDF membrane. The membrane was blocked with 5% BSA in TBST containing 50 mM Tris–HCl, 150 mM NaCl, and 0.1% Tween-20 and incubated with primary antibody solutions, phospho-JNK (1:1000; Cell Signaling, Cat# 9251S), JNK (1:1000; Cell Signaling, Cat# 9252S), phospho-p38 MAPK (1:1000; Cell Signaling, Cat# 9211S), p38 MAPK (1:1000; Cell Signaling, Cat# 9212S), phospho-MEK1/2 (1:1000; Cell Signaling, Cat# 9154S), MEK1/2 (1:1000; Cell Signaling, Cat# 9122S), phospho-KF-kB p65 (1:1000; Cell Signaling, Cat# 3033S), NF-kB p65 (1:1000; Cell Signaling, Cat# 8242S), phospho-SEK1/MKK4 (1:1000; Cell Signaling, Cat# 9156S), SEK1/MKK4 (1:1000; Cell Signaling, Cat# 9152S), Phospho-PAK1 (1:1000; Cell Signaling, Cat# 2601S), PAK1 (1:1000; Cell Signaling, Cat# 2608S), phospho-p44/42 MAPK (ERK1/2) (1:1000; Cell Signaling, Cat# 9101S), p44/42 MAPK (ERK1/2) (1:1000; Cell Signaling, Cat# 9102S), and HA antibody (1:1000; BioLegend, Cat# 901502) diluted in a blocking buffer overnight at 4 °C. Appropriate HRP-conjugated secondary antibodies were used to visualize the proteins by chemiluminescence. Blots were analyzed using the iBright imaging system. For the JNK inhibitor effect analysis, cells were incubated in 25 mM or 5 mM glucose with or without JNK inhibitor (Santa Cruz Biotechnology, Cat# sc-200635) (1 μM for migration assay and 4 μM for phosphorylation analysis) for 6 h.

### Co-immunoprecipitation

For co-immunoprecipitation of PP2Cα and its substrates (JNK, ERK1/2, and MEK4), MDA-MB-231 cells were transfected with HA-PP2Cα-WT or C314S plasmid. After 24 h, cells were incubated in 25 mM or 5 mM glucose in glucose-free DMEM for 6 h. Cells were then lysed with a mild lysis buffer containing 1% NP-40, 150 mM NaCl, and 50 mM Tris-pH 8. One milligram of lysate was incubated with HA antibody (3 μl) for 1 h at 4 °C. The solution was mixed with prewashed protein G agarose beads (25 μl beads or 50 μl with slurry) and incubated overnight at 4 °C. Beads were washed three times with 500 μl TBST (50 mM Tris–HCl, 150 mM NaCl, and 0.1% Tween-20) for 10 min each. Proteins on beads were eluted by adding SDS-loading dye (50 μl × 2) containing β-mercaptoethanol (3 μl) and heating at 95 °C for 10 min. Eluted proteins were resolved by SDS-PAGE and transferred to the PVDF membrane. The membrane was blocked with 5% BSA in TBST (50 mM Tris–HCl, 150 mM NaCl, and 0.1% Tween-20) and incubated with primary antibody solutions, such as JNK (1:1000), ERK1/2 (1:1000), MAP2K4/MEK4 (1:1000), or HA antibody (1:1000), diluted in a blocking buffer overnight at 4 °C. Appropriate HRP-conjugated secondary antibodies were used to visualize the proteins by chemiluminescence. Blots were analyzed using the iBright imaging system (Thermo Fisher Scientific).

### Preparation of KO cell line

MCF7 cells were transfected with pSp-Cas9(BB)-2A-GFP-sgRNA constructs with Lipofectamine 3000. Seven micrograms from each pSP-Cas9 (BB)-2A-GFP-sgRNA (sgRNA1 and sgRNA2) was used with 21 μl Lipofectamine and 28 μl P3000 reagent. After 48 h, cells were sorted in groups by FACS into a 6-well plate containing 1 ml DMEM with 10% FBS. After growing for 2 weeks, cells were subjected to single-cell sorting using FACS. Single cells were sorted into each well in 96-well plates (n = 5) containing 100 μl DMEM with 10% FBS. Cells were grown until confluent. Cells produced by a single colony were picked (∼50 colonies) and transferred to 12-well plates. When cells were confluent, they were transferred to the 6 cm dish. After the 6 cm dish became confluent, cell stocks were prepared. Lysates were collected and subjected to western blotting with PP2Cα primary antibody (1:1000).

### Statistical analysis

All data are shown with the means ± SD and were statistically analyzed by one-way ANOVA followed by Tukey’s *post hoc* test, two-way ANOVA followed by Sidak’s or Tukey’s *post hoc* test, or Student’s *t* test with Welch’s correction. The value *p* < 0.03 is statistically significant.

## Data availability

All data supporting the findings of this study are available from the corresponding author upon request.

## Supporting information

This article contains supporting information.

## Conflicts of interests

The authors declare that they have no conflicts of interest with the contents of this article.
