# Supplementary material for: Protein phosphatase PP2Cα S-glutathionylation regulates cell migration
Source: J Biol Chem. 2024 Sep 18;300(10):107784. doi: 10.1016/j.jbc.2024.107784 (PMC11530597; doi:10.1016/j.jbc.2024.107784)
Supplement: Supplemental Information [file mmc1.pdf]

Supporting Information

Protein phosphatase PP2Cα S-glutathionylation regulates cell migration

DhDhanushika S. K. Kukulage,[1] Kusal T. G. Samarasinghe,[2] Nadee N. J. Matarage Don,[1] Madhu C. Shivamadhu,[1] Kyosuke Shishikura,[3] William Schiff,[1] Faezeh Mashhadi Ramezani,[1] Rayavarapu Padmavathi,[1] Megan L. Matthews,[3] and Young-Hoon Ahn[1]*

List of Supplemental Figures

Figure S1. Migration analyses of MCF7 and MDA-MB-231 cell lines in low glucose or by DAAO.

Figure S2. Clickable glutathione approach with mass spectrometric analysis of glutathionylated cysteines

Figure S3. Proteins and cysteines susceptible to glutathionylation and involved in cell migration.

Figure S4. Screening of 9 selected proteins for their cell migration in low and high glucose conditions

Figure S5. Migration analysis of 3 proteins in MCF7 in low and high glucose conditions

Figure S6. Structure and sequence analyses of PP2Cα

Figure S7. Analysis of PP2Cα glutathionylation in vitro and in cells

Figure S8. Mass analysis of PP2Cα glutathionylation

Figure S9. Migration and invasion of MDA-MB-231 upon PP2Cα glutathionylation

Figure S10. Single-cell migration tracking analysis

Figure S11. PP2Cα glutathionylation and cell migration induced by PROX-D

Figure S12. Enzyme kinetic analysis of PP2Cα with and without glutathionylation

Figure S13. Analysis of PP2Cα interacting proteins

Figure S14. Mechanistic analysis of PP2Cα glutathionylation with its downstream substrates

Figure S15. Preparation and evaluation of MCF7 PP2Cα KO cell line for cell migration

Figure S16. Single-cell migration tracking analysis of MCF7 PP2Cα KO cell line

Figure S17. EGF induces PP2Cα glutathionylation at C314

Supplementary Methods

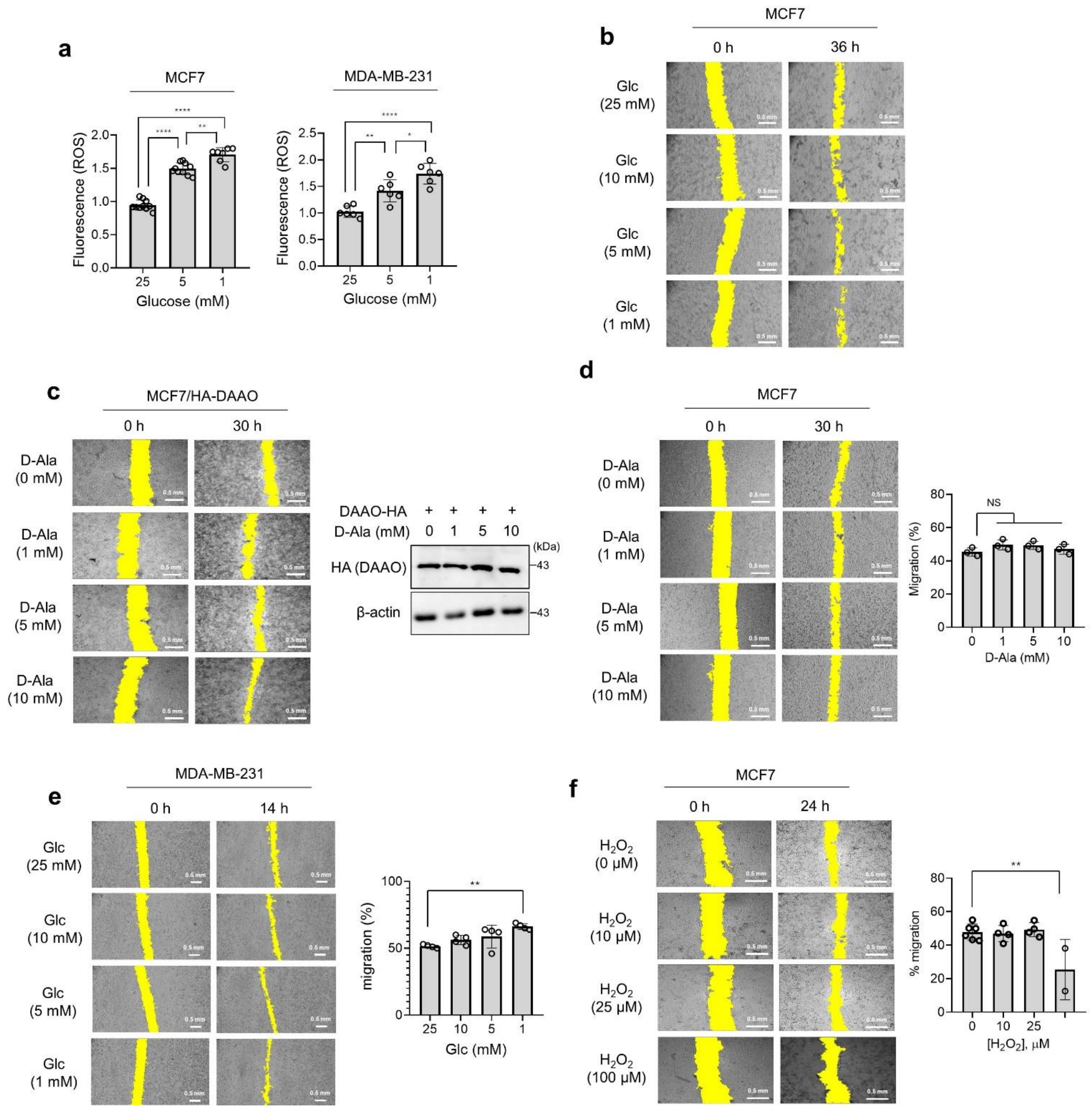

Supplementary Figure 1. ROS levels and migration analyses of MCF7 and MDA-MB-231 cell lines in low glucose or by DAAO. (**a**) ROS levels in MCF7 and MDA-MB-231 cells in different concentrations of glucose. Cells were incubated in DMEM with indicated concentrations of glucose for 24 h, and ROS levels were measured using 2',7'-dichlorodihydrofluorescein diacetate (DCF-DA) probe (n = 3). (**b**) Migration of MCF7 cells in different concentrations of glucose (representative images of Figure 1A, n = 3). (**c**) Migration of MCF7 cells expressing DAAO upon adding D-Ala (representative images of Figure 1B, n = 4). (**d**) Migration of MCF7 cells upon adding D-Ala (n =3). (**e**) Migration of MDA-MB-231 in different concentrations of glucose. (**f**) Migration of MCF7 upon a bolus addition of H_2O_2 (n =3). Migration was measured by wound healing assay. The yellow color in (**b**-**f**) indicates the area without cells. Data represents the mean ± SD. The statistical difference was analyzed by one-way ANOVA and Tukey's post-hoc test, where *p < 0.03, **p < 0.002, ***p < 0.0002, ****p < 0.0001. The scale bar in (**b-f**) = 0.5 mm.

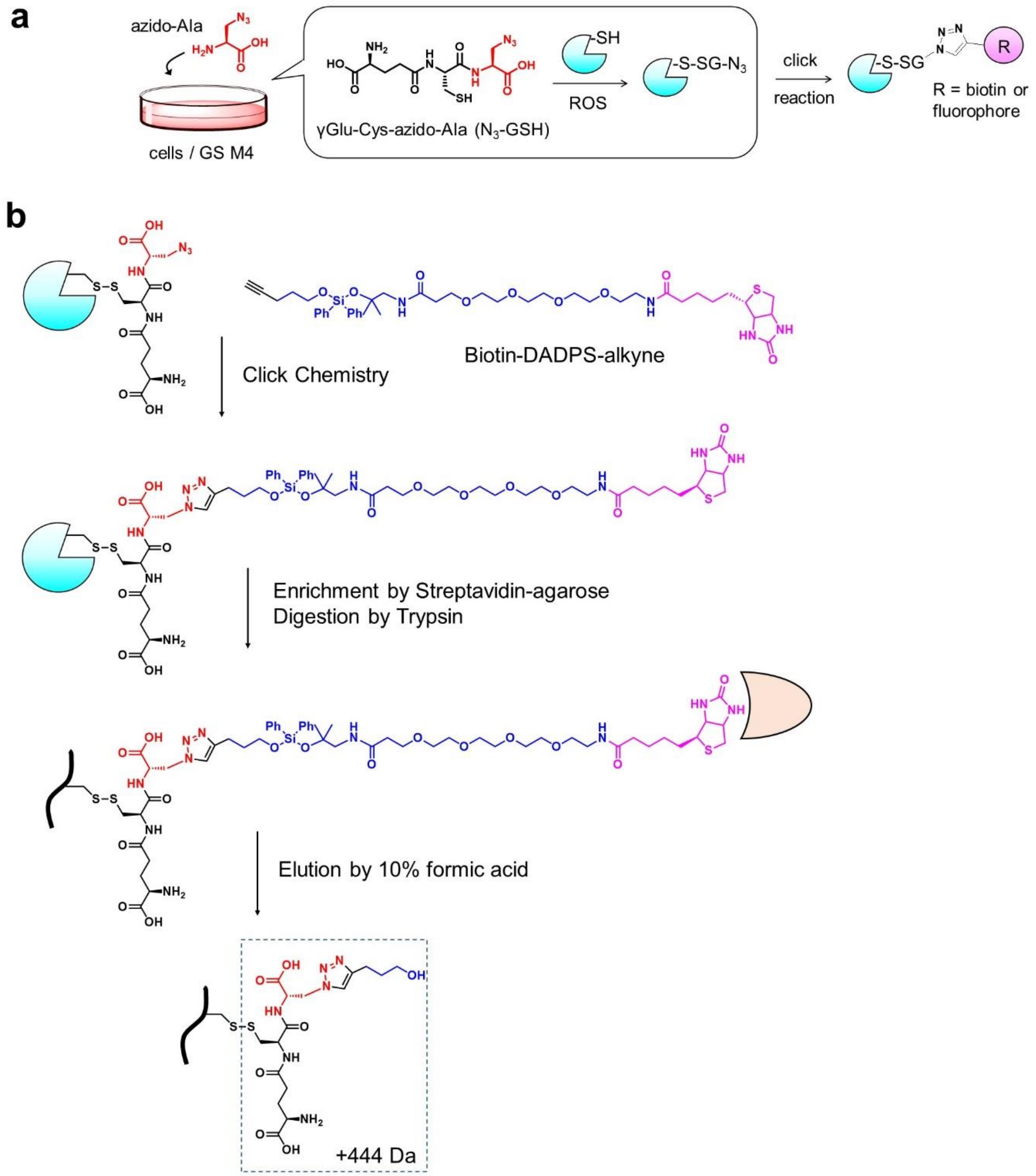

Supplementary Figure 2. Clickable glutathione approach with mass spectrometric analysis of glutathionylated cysteines. (**a**) Clickable glutathione approach. Cells expressing glutathione synthetase mutant (GS M4) are incubated with azido-Ala, synthesizing clickable azido-glutathione (N_3-GSH). Glutathionylated proteins by N_3-GSH are analyzed after click chemistry with fluorophore- or biotin-alkyne. (**b**) The mass spectrometric analysis of glutathionylated cysteines. Glutathionylated proteins (purified or in lysates) are conjugated with biotin-DADPS-alkyne, enriched by streptavidin-agarose, and digested by trypsin. Glutathionylated peptides are then eluted in an acidic solution while retaining the glutathione modification on the cysteine sites, which are analyzed by LC-MS/MS.

a

Kinase	
**Protein**	**Cys for SSG**
**CSK**	C31, C290
**MAPK9**	C177
**STK10**	C887
Coq8B	C148, C336

Phosphatase	
**Protein**	**Cys for SSG**
**PP2Cα**	C314
PTPRU	C1380

GTPase Activating Protein	
**Protein**	**Cys for SSG**
ARHGAP18	C489
SRGAP2	C338, C357
RABGAP1L	C248
RAB3GAP1	C873, C106

Guanine Nucleotide Exchange Factor	
**Protein**	**Cys for SSG**
**ARHGEF7**	C700, C469
DOCK4	C860

Cytoskeletal Proteins	
**Protein**	**Cys for SSG**
**CFL1**	C39, C80, C139, C147
NEXN	C569, C149
WDR1	C225
VASP	C332
FLNA	C8, C478, C483, C574, C717, C841, C1018, C1157, C1260, C1312, C1912, C1920, C2199
ANLN	C232, C328, C348, C816, C1004, C1010
PDLIM1	C73
FLNB	C1081, C1155, C1158, C1434

Other Proteins	
**Protein**	**Cys for SSG**
**RAB21**	C27
**NISCH**	C186
**MGLL**	C201,C208, C301
LAMC1	C1598
MIF	C81
DPYSL2	C504
SUN1	C501, C635, C759
STOML2	C167
CPNE3	C506
SCRIB	C1113
WDR26	C218
FLII	C46
PTBP3	C39
TACC2	C1060
NUP93	C522, C422
RTN4	C319, C537, C884, C889
PLCG1	C976

b

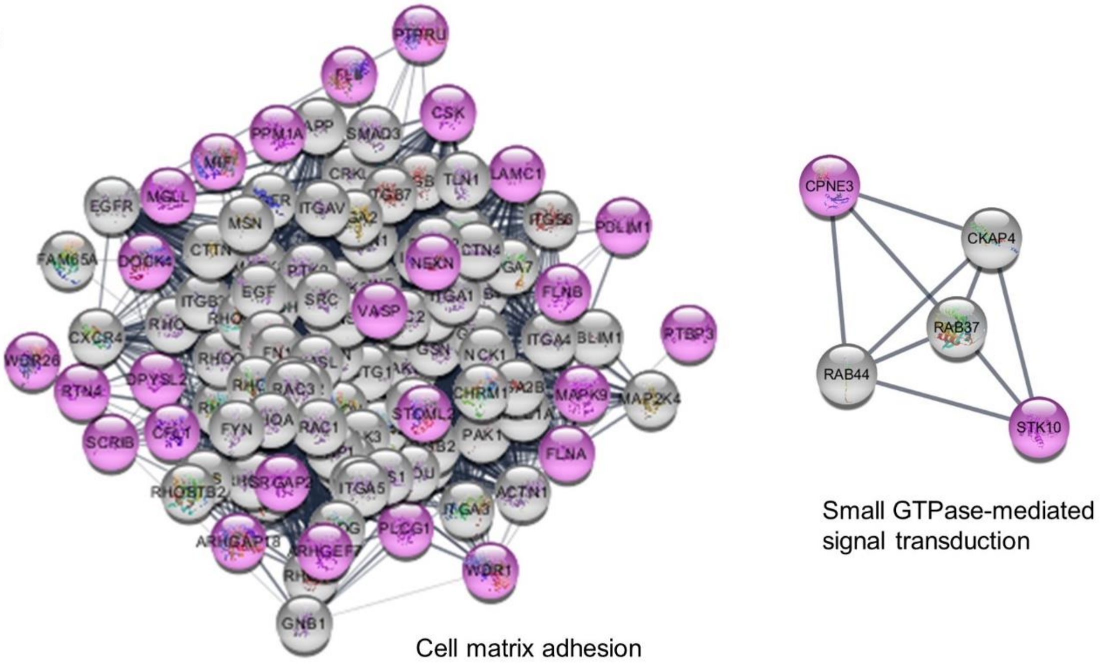

Supplementary Figure 3. Proteins and cysteines susceptible to glutathionylation and involved in cell migration. (**a**) 37 proteins and 74 cysteines belonging to both groups of “proteins for glutathionylation” (n = 1,351) and “proteins in migration” (n = 536). Proteins were analyzed in Cytoscape and classified by their protein functions. All cysteine numbers are from mouse, except PP2Cα C314 from human. (**b**) The cluster analysis of 37 identified proteins in (**a**) with their interacting proteins (n = 50) in Cytoscape. Their biological functions were analyzed using DAVID GO analysis.

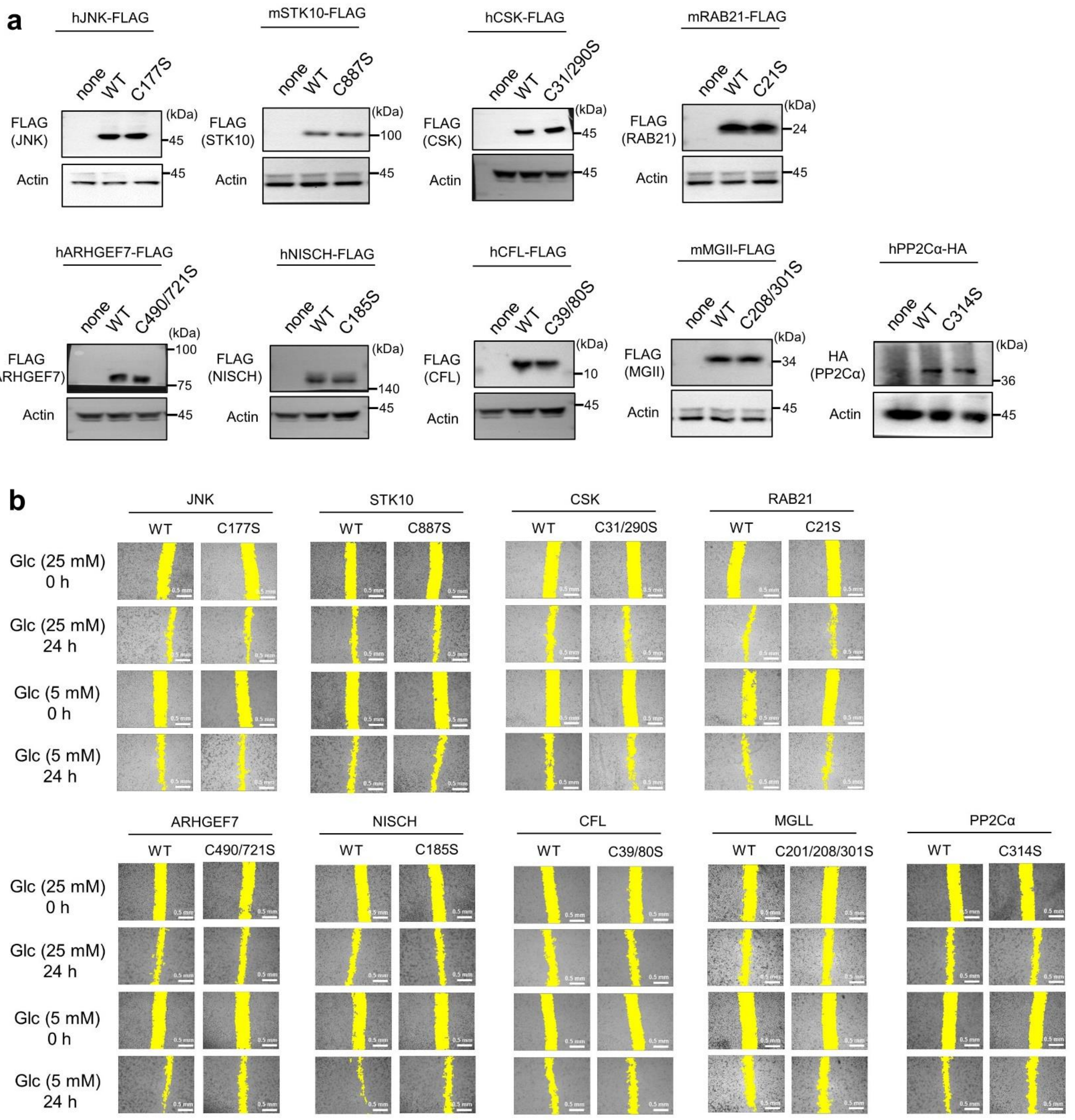

Supplementary Figure 4. Biological screening of 9 selected proteins for their cell migration in low and high glucose conditions. MDA-MB-231 cells were transfected with WT or cysteine mutant (C/S) of 9 selected proteins. Cell migration was analyzed in low (5 mM) or high (25 mM) glucose by the wound healing assay. (**a**) Western blots showing expression of WT and C/S of 9 proteins. WT and C/S were transfected for 48 h. (**b**) Representative image of migration analyses of cells expressing individual protein WT or C/S (representative images of Figure 1F, n = 3). The yellow color in (**b**) indicates the area without cells. The scale bar = 0.5 mm. The gene sequences for STK10, Rab21, and MGLL are from mouse and all others from human.

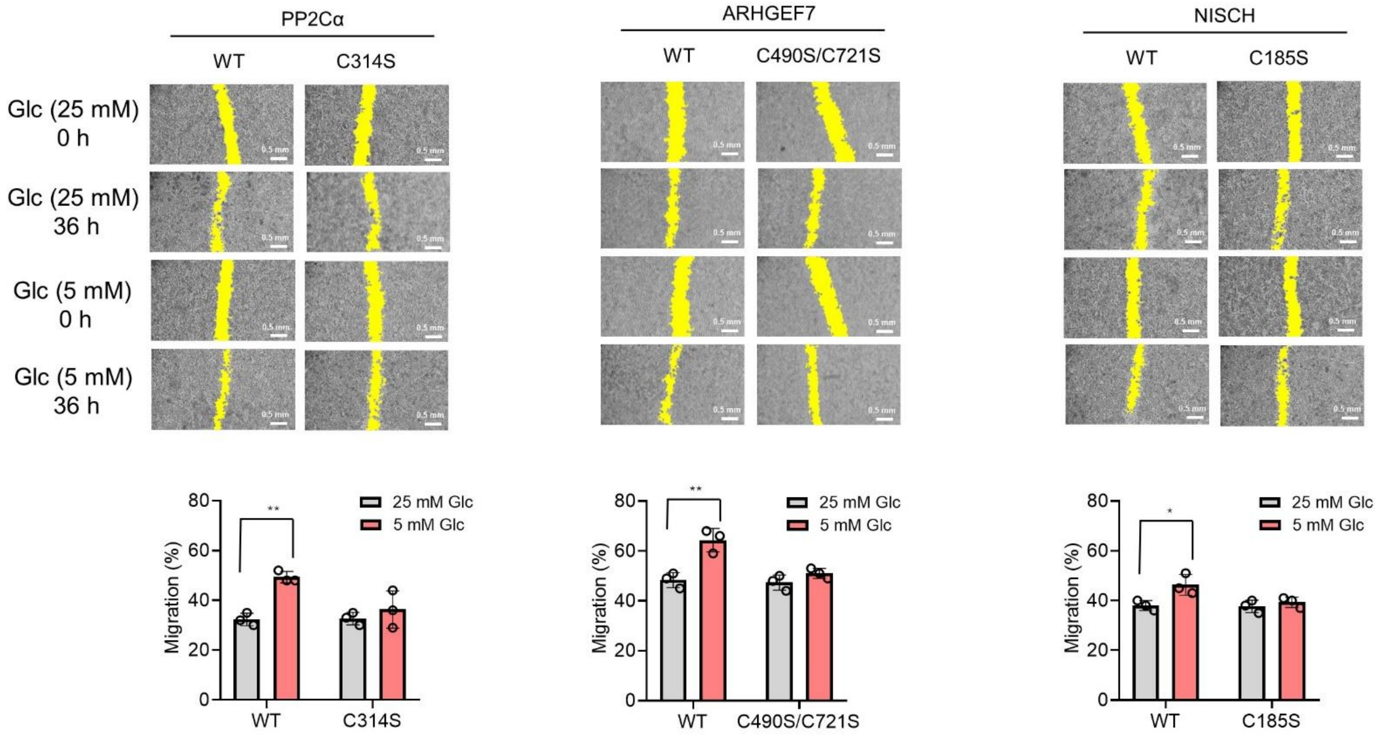

Supplementary Figure 5. Migration analysis of 3 selected proteins in MCF7 in low and high glucose conditions. MCF7 cells were transfected with WT or cysteine mutant (C/S) of 3 selected proteins (PP2Cα, ARHGEF7, and NISCH). After 48 h, cells were incubated in low (5 mM) or high (25 mM) glucose for 36 h. Cell migration was analyzed by the wound healing assay. Representative image of migration analyses of cells expressing individual protein WT or C/S (top) and quantification analysis (bottom) (n = 3). The yellow color in the images indicates the area without cells. The scale bar = 0.5 mm. Data represents the mean ± SD. The statistical difference was analyzed by two-way ANOVA and Tukey's post-hoc test, where $*p < 0.03$, $**p < 0.002$, $***p < 0.0002$, $****p < 0.0001$.

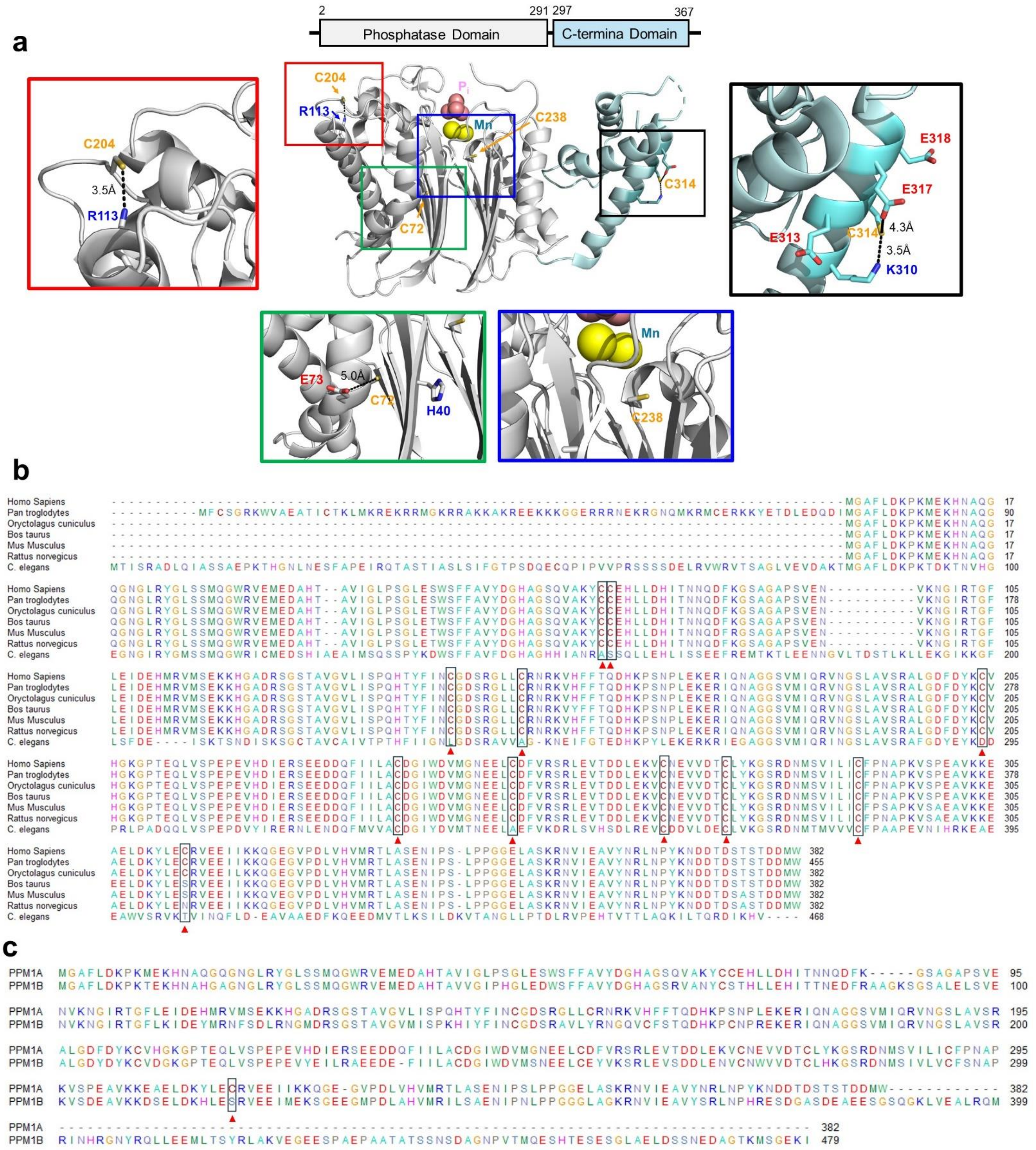

Supplementary Figure 6. Structure and sequence analyses of PP2Cα. (**a**) PP2Cα structure with cysteines. C72, C204, and C238 are located in the catalytic domain, while C314 is positioned in the C-terminal domain (CTD). Phosphate and Mn^{2+} ions in the active sites are shown in sphere. The cysteines and their neighboring amino acids within 5 Å distance are shown by a stick model. (**b**) Sequence alignment of PP2Cα in humans and other species. Human PP2Cα cysteines are boxed with their aligned ortholog amino acids. (**c**) Sequence alignment of PP2Cα (PPM1A) with its paralog PP2Cβ (PPM1B). PP2Cα C314 position is boxed.

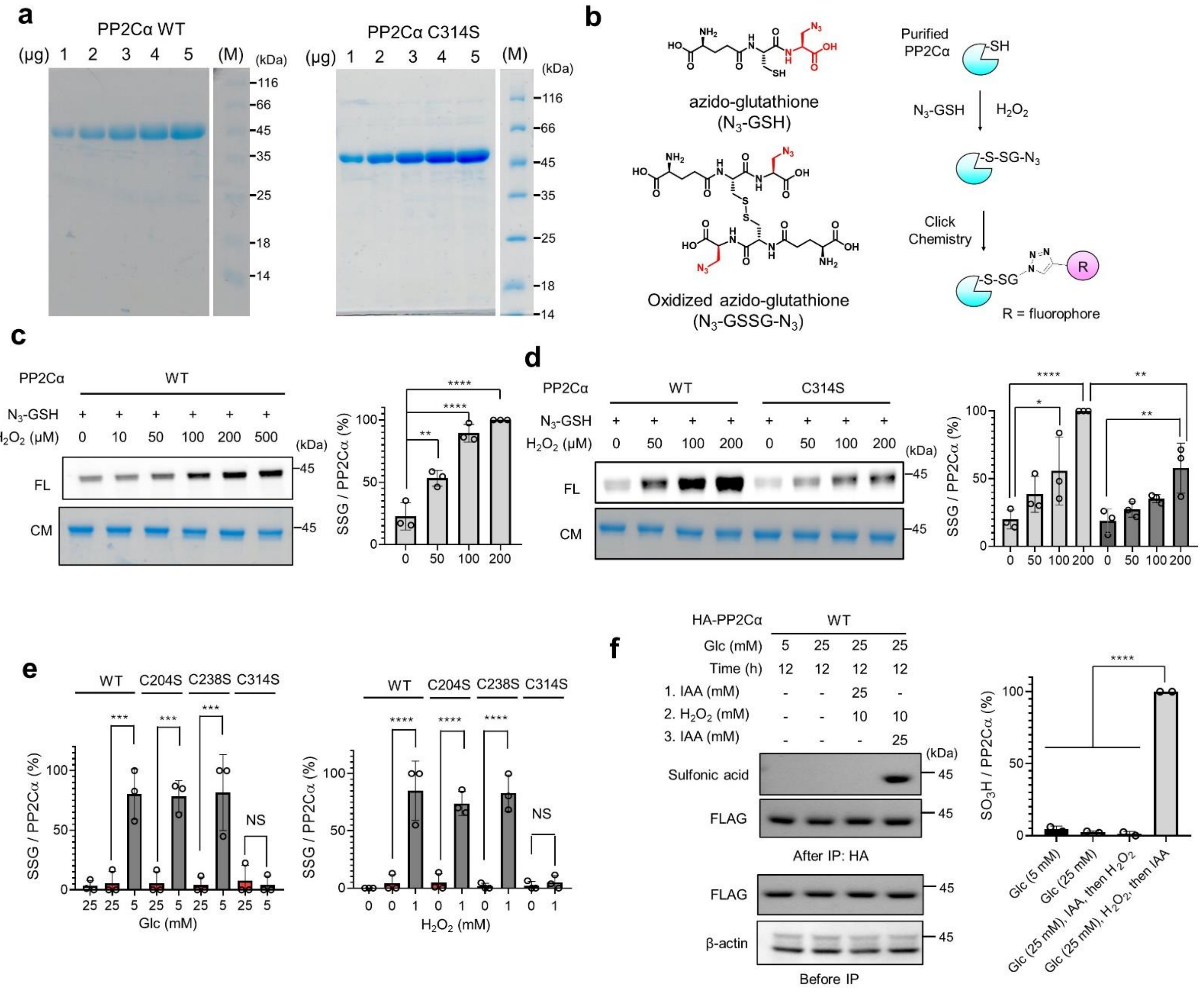

Supplementary Figure 7. Analysis of PP2Cα glutathionylation in vitro and in cells. (**a**) Purified PP2Cα WT and C314S. (**b**) Structures of azido-glutathione (N_3-GSH) and its oxidized form (N_3-GSSG-N_3) (left). PP2Cα reaction with N_3-GSH (right). (**c**-**d**) PP2Cα glutathionylation in vitro. Purified PP2Cα WT and C314S in the presence of N_3-GSH were incubated with H_2O_2 for 15 min and analyzed after click reaction with rhodamine-alkyne via fluorescence (FL, SSG level) and Coomassie stain (CM, protein level). The representative gel images (n = 3) are shown. (**e**) Quantification analysis of PP2Cα glutathionylation in cells (data analysis of Figure 2H, n = 3). (**f**) PP2Cα analysis for sulfinic or sulfonic acid modification. MDA-MB-231 cells expressing PP2Cα WT were incubated in low or high glucose for 12 h. Lysates were treated in a sequence of #1-3. PP2Cα was immune-precipitated and analyzed by western blot with antibodies against sulfonic acid (SO_3H antibody). Lanes 3 and 4 are negative and positive controls for SO_3H antibody, respectively, showing that the antibody appears to recognize over-oxidized cysteines in PP2Cα oxidized by a large amount of H_2O_2 (10 mM). Lanes 1-2 show no detection of sulfonic acid or over-oxidation forms of cysteine in PP2Cα in low or high glucose (n = 3). Data represents the mean ± SD. The statistical difference was analyzed by one-way ANOVA and Tukey's post-hoc test, where $*p < 0.03$, $**p < 0.002$, $***p < 0.0002$, $****p < 0.0001$.

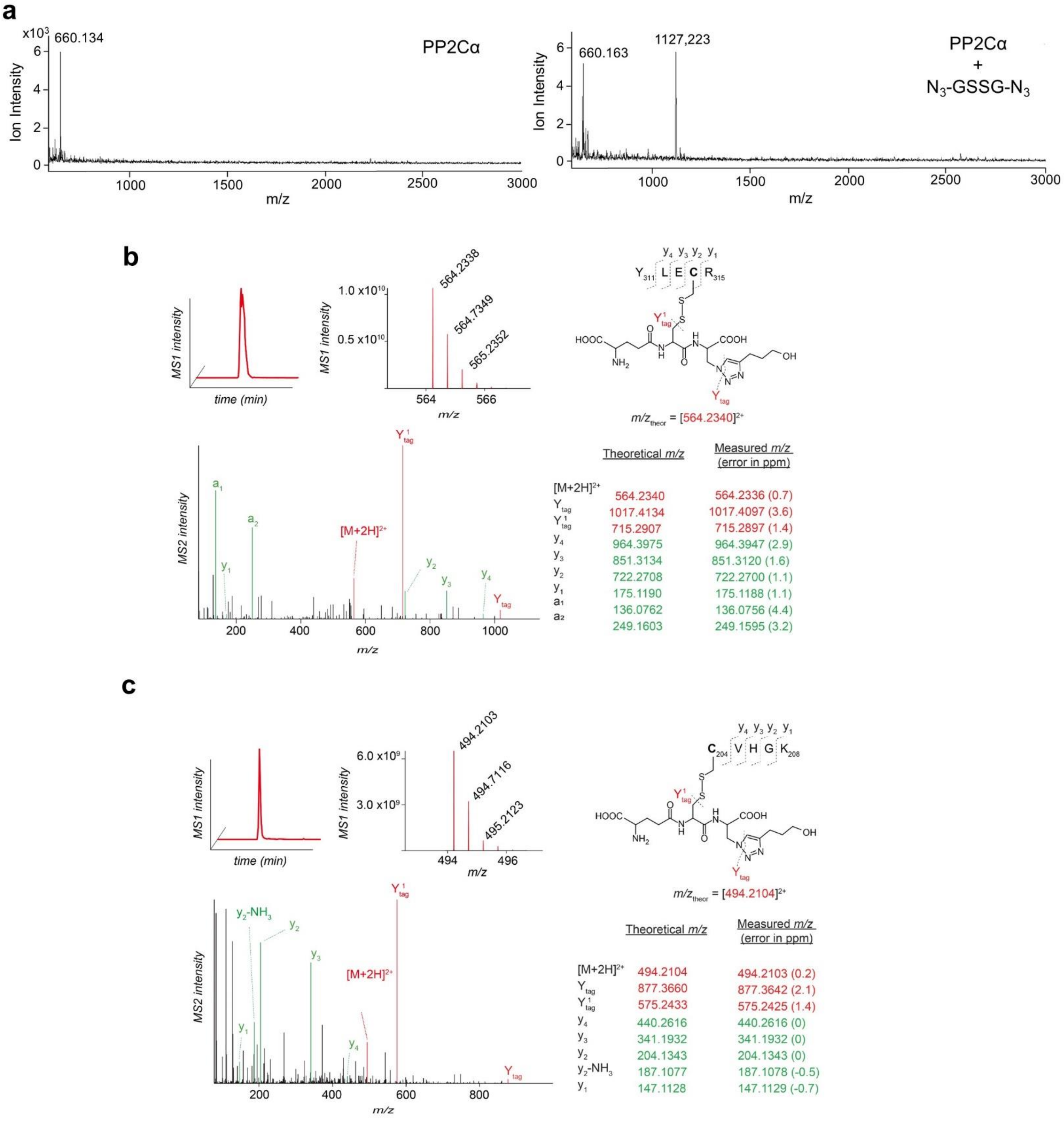

Supplementary Figure 8. Mass analysis of PP2Cα glutathionylation. a. MALDI-TOF analysis of glutathionylated PP2Cα using a clickable glutathione approach outlined in Supplementary Figure 2b. Purified PP2Cα was glutathionylated by incubating none (left) or N_3-GSSG-N_3 (right) for 2 h. After click reaction with biotin-DADPS-alkyne, pull-down by streptavidin-agarose, and on-bead trypsin digestion, glutathionylated peptide was eluted and analyzed by MALDI-TOF, which found one main peak corresponding to a peptide glutathionylated at C314, YLEC$_{314}$*R (calculated m/z 1127.461, found m/z 1127.223) (n = 3). **b**-**c**. LC-MS/MS analysis of glutathionylated PP2Cα. The eluted sample was further analyzed by LC-MS/MS, confirming the

peptide glutathionylated at C314 (**b**) and finding a peptide glutathionylated at C204 (**c**). Both **b** and **c** contain the extracted parent ion chromatograms and corresponding isotopic envelopes (top left), structures and parent masses of glutathionylated peptides (top right), MS2 spectra (bottom left), and a table of theoretical and measured spectra assignment in high-resolution MS2 conditions (bottom right).

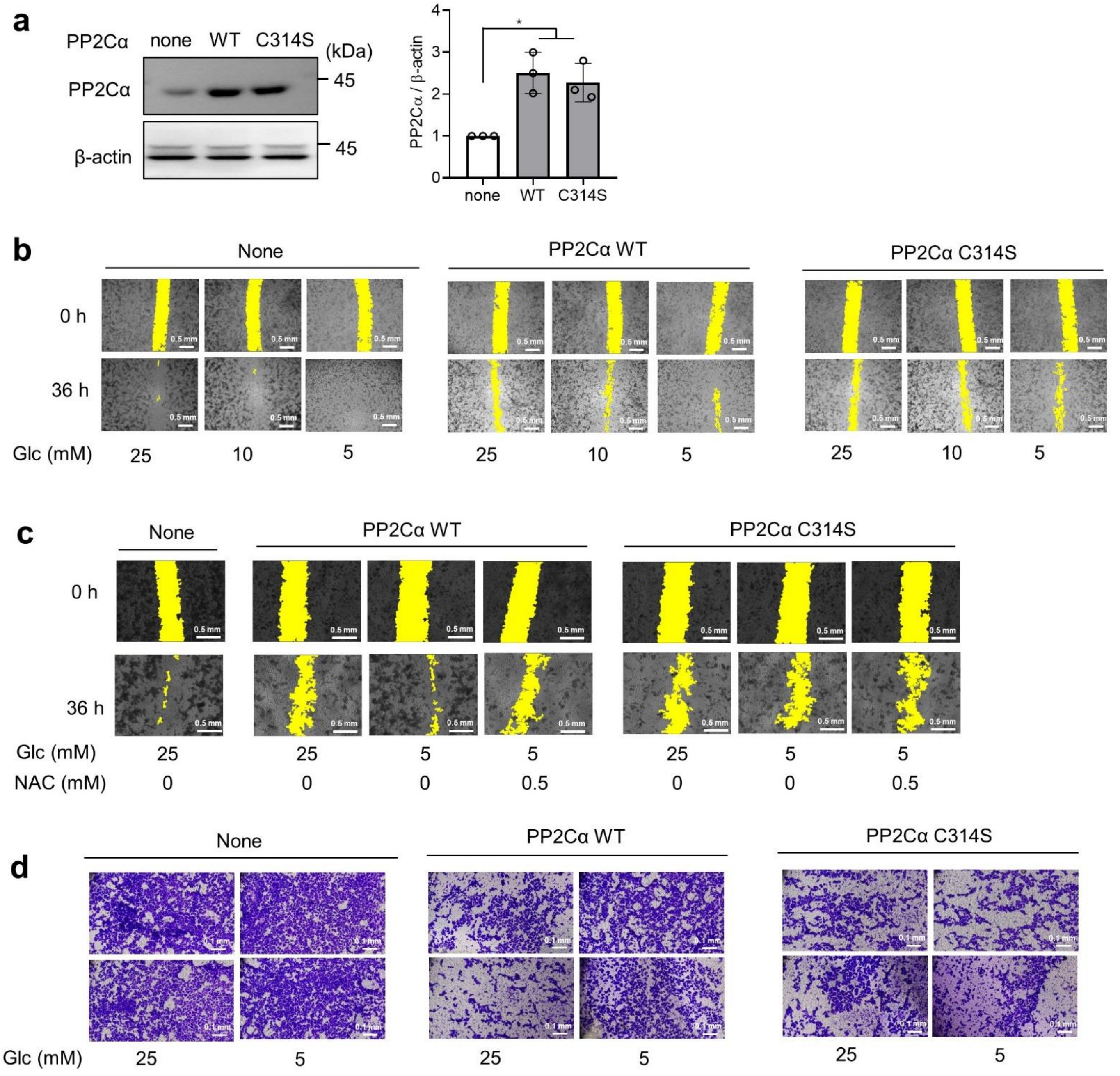

Supplementary Figure 9. Migration and invasion of MDA-MB-231 upon PP2Cα glutathionylation. (**a**) PP2Cα expression level without (none) or with transfection of WT or C314S for 24 h (n = 3). (**b**) Migration of cells with PP2Cα WT or C314S in high or low glucose (representative images of Figure 3A, n = 3). (**c**) Cell migration in high or low glucose with the addition of N-acetylcysteine (NAC) (representative images of Figure 3C, n = 3). The yellow color in (**b**-**c**) indicates the area without cells. The scale bar in (**b**-**c**) = 0.5 mm. (**d**) Transwell invasion of cells with PP2Cα WT or C314S in high or low glucose (representative images of Figure 3B, n = 3). The scale bar = 0.1 mm. Data represents the mean ± SD. The statistical difference was analyzed by one-way ANOVA and Tukey's post-hoc test, where *p < 0.03, **p < 0.002, ***p < 0.0002, ****p < 0.0001.

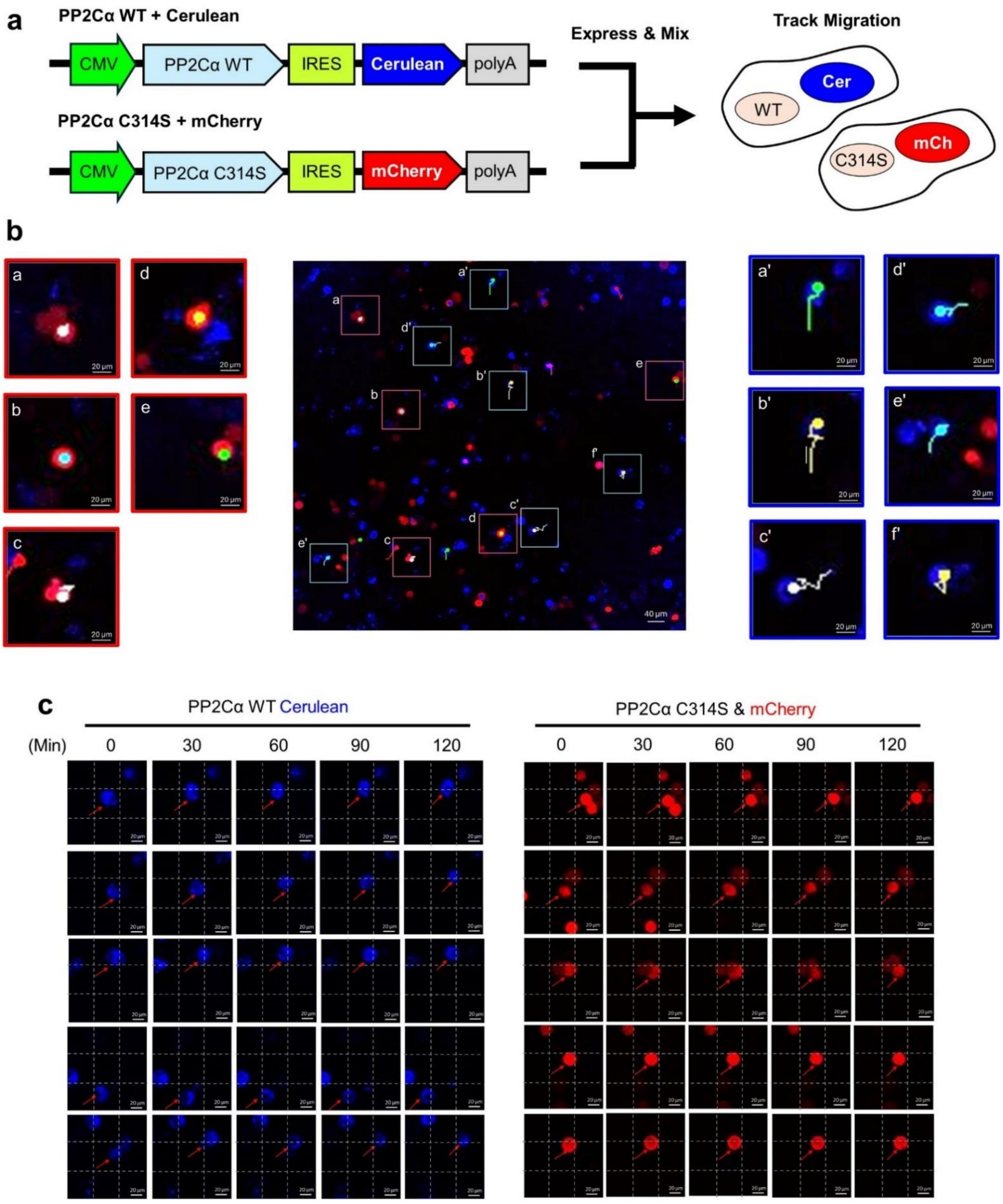
a
PP2Cα WT + Cerulean
CMV
PP2Cα WT
IRES
Cerulean
polyA
PP2Cα C314S + mCherry
CMV
PP2Cα C314S
IRES
mCherry
polyA
Express & Mix
Track Migration
Cer
WT
mCh
C314S
b
c
PP2Cα WT Cerulean
PP2Cα C314S & mCherry
(Min)
0
30
60
90
120

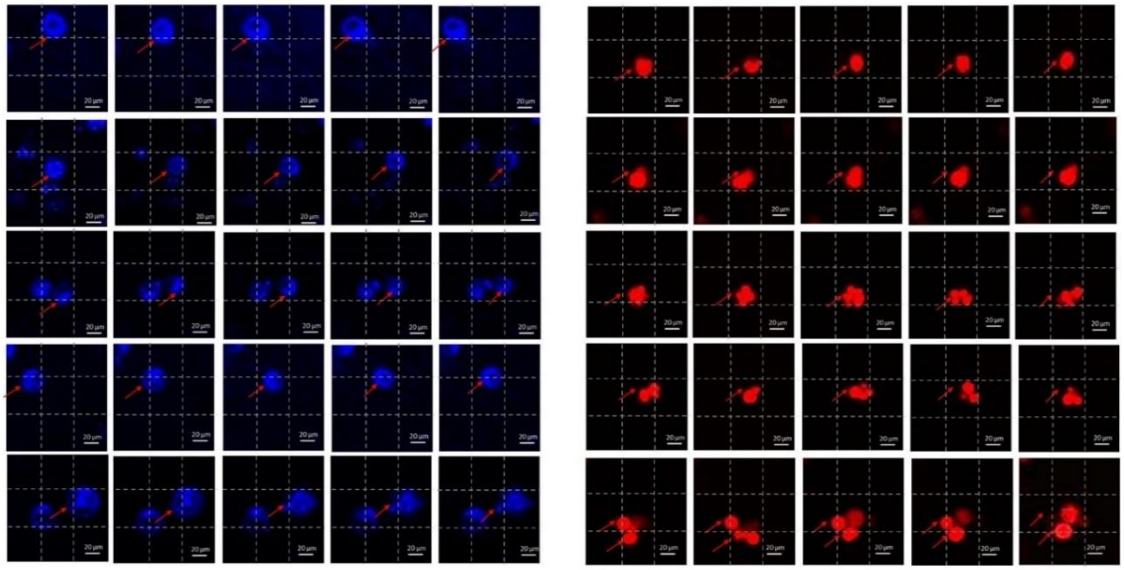

d

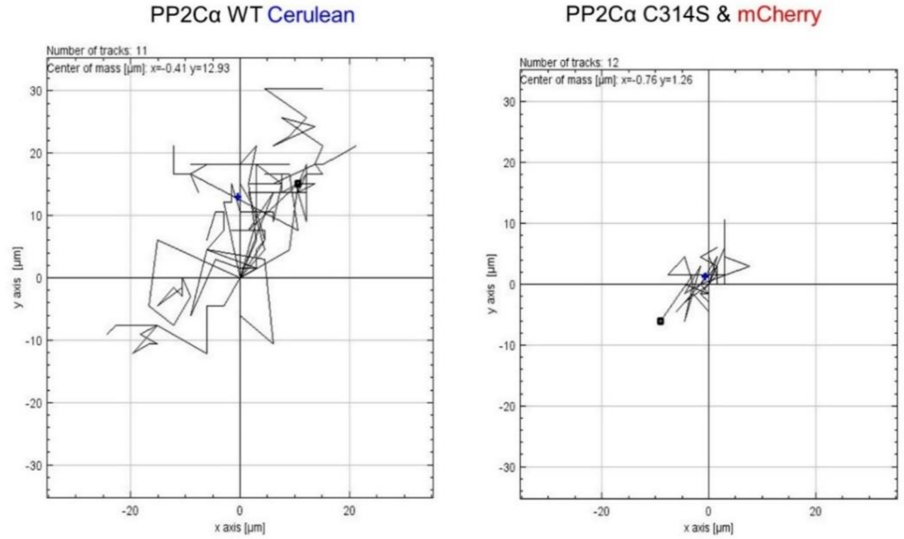

Supplementary Figure 10. Single-cell migration tracking analysis. (**a**) A scheme for single cell migration analysis. Bicistronic plasmids were transfected to MDA-MB-231 cells to express PP2Cα WT and Cerulean (PP2Cα WT-Cerulean) or express PP2Cα C314S and mCherry (PP2Cα C314S-mCherry). Two groups of cells were combined, and individual cells were monitored for migrations via fluorescence confocal microscopy. (**b**) Migration traces of individual cells. Cells were incubated in low glucose (5 mM) with EGF for 2 h (migration was not apparent without EGF in 2 h). The images were taken every 10 min and analyzed by the Image-J (Manual tracking and chemotaxis tools), which shows the migration traces (tails), migration velocity, and distances. A low magnification image (center) and the selected individual cells (left and right). The image in Figure 3f was re-used in the center of Figure 10b. Red cells show a dot as a migration trace indicating limited migration, whereas blue cells show a tail as a trace indicating their migration path and distance. The scale bar = 20 µm (left and right) or 40 µm (middle). (**c**) Representative images of 10 cells in blue or red. Images in Figure 3g are re-shown in Figure 10c. The scale bar = 20 µm. (**d**) The chemotaxis analysis. The original positions of individual cells are centered with their migration tracks drawn in 2-D plots, showing stochastic migration directions with longer migration tracks by PP2Cα WT versus C314S. The scale bar = 20 µm.

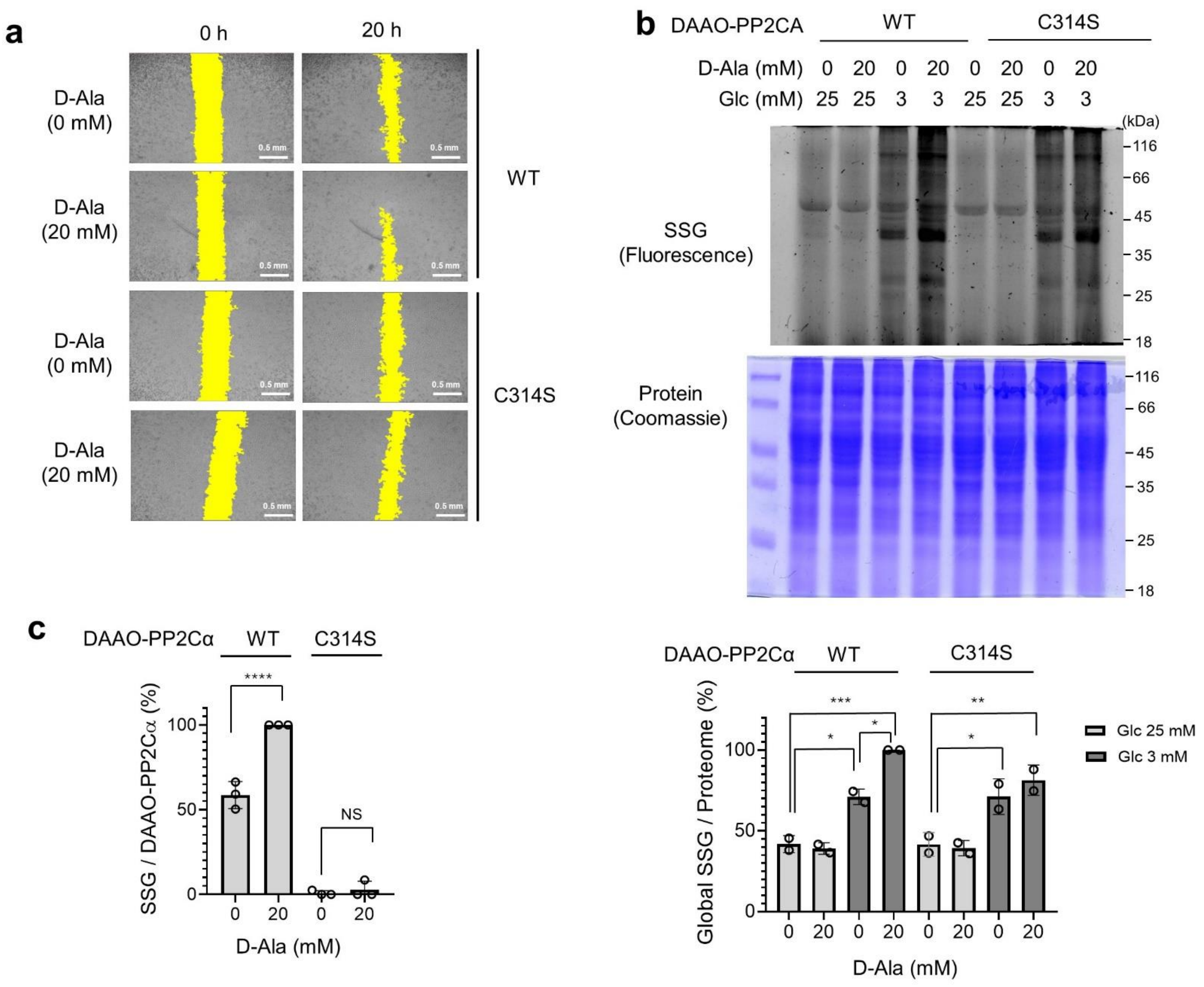

Supplementary Figure 11. PP2Cα glutathionylation and cell migration induced by PROX-D. MDA-MB-231 cells expressing DAAO-PP2Cα WT or C314S were incubated with D-Ala for 20 h. (**a**) Cell migration induced by PROX-D (representative migration images, n = 3). The quantification analysis is shown in Figure 3J. The yellow color in (**a**) indicates the area without cells. The scale bar = 0.5 mm. (**b**) Global glutathionylation induced by a DAAO-PP2Cα/D-Ala approach. Lysates were click-conjugated with rhodamine-alkyne and analyzed by fluorescence (SSG level) and Coomassie stain (protein level). In the presence of high glucose (25 mM), the addition of D-Ala (up to 20 mM) did not cause significant changes in glutathionylation (lanes 1-2 and 5-6). Therefore, glutathionylation induced by D-Ala was evaluated in low glucose (3 mM), which causes the elevation of global glutathionylation (lanes 3-4 and 7-8) (n = 2). (**c**) PP2Cα glutathionylation induced by PROX-D (quantification analysis of Figure 3K, n = 3). Data represents the mean ± SD. The statistical difference was analyzed by one-way ANOVA and Tukey's post-hoc test, where *p < 0.03, **p < 0.002, ***p < 0.0002, ****p < 0.0001.

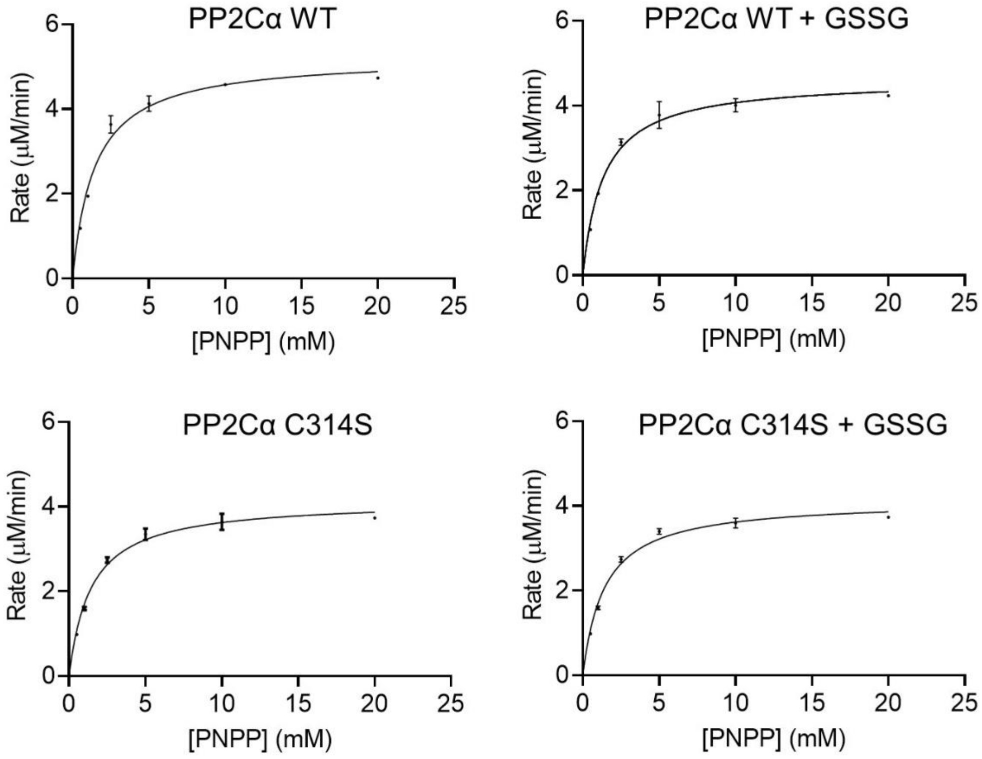

Supplementary Figure 12. Enzyme kinetic analysis of PP2Cα with and without glutathionylation. Purified PP2Cα WT or C314S was incubated without or with GSSG for 2 h. PP2Cα enzyme activity was then measured using its substrate, p-nitrophenyl phosphate (pNPP) (n = 2). Data were measured in duplicate and shown with the mean ± SD. Non-linear regression curves were produced by fitting data to the Michaelis-Menten equation using Prism software.

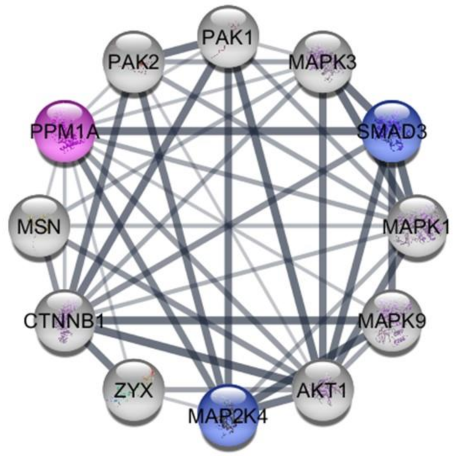

Supplementary Figure 13. Analysis of PP2Cα interacting proteins. The neighboring proteins of PP2Cα were found in the STRING database.

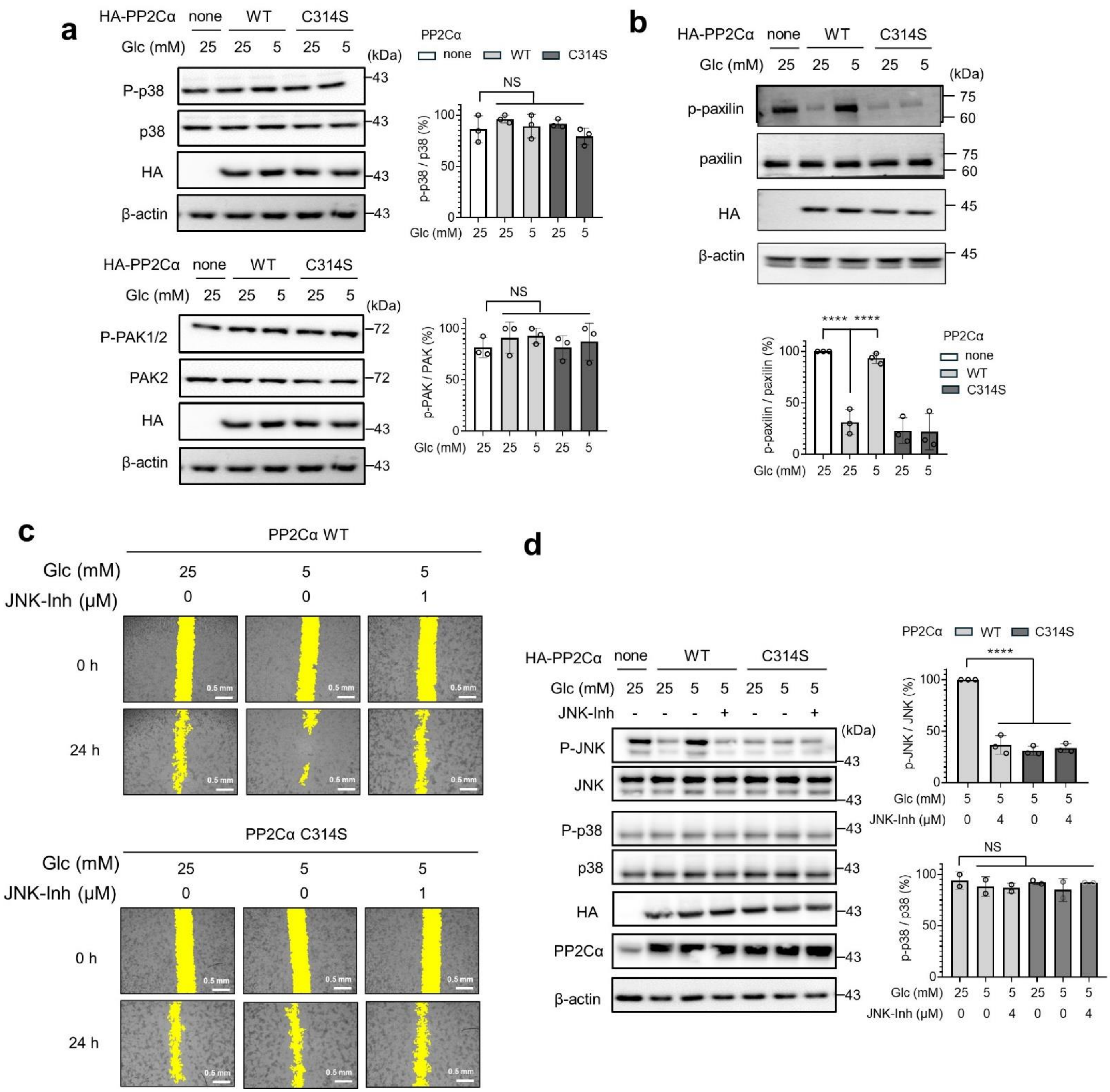

Supplementary Figure 14. Mechanistic analysis of PP2Cα glutathionylation with its downstream substrates. (**a**) Phosphorylation states of p38 and PAK upon PP2Cα glutathionylation (n = 3). (**b**) Phosphorylation states of paxillin upon PP2Cα glutathionylation. MDA-MB-231 cells expressing PP2Cα WT or C314S in low or high glucose for 6 h, followed by western blot analyses (n = 3). (**c**-**d**) JNK inhibitor reduces cell migration induced by PP2Cα glutathionylation. Cell migration assay in low or high glucose upon adding JNK inhibitor (1 μM) (representative images of Figure 4B) (**c**). The effect of JNK inhibitor (4 μM) on the phosphorylation state of JNK and p38 (n = 3) (**d**). The yellow color in (**c**) indicates the area without cells. The scale bar in (**c**) = 0.5 mm. Data represents the mean ± SD. The statistical difference was analyzed by one-way ANOVA and Tukey's post-hoc test, where *p < 0.03, **p < 0.002, ***p < 0.0002, ****p < 0.0001.

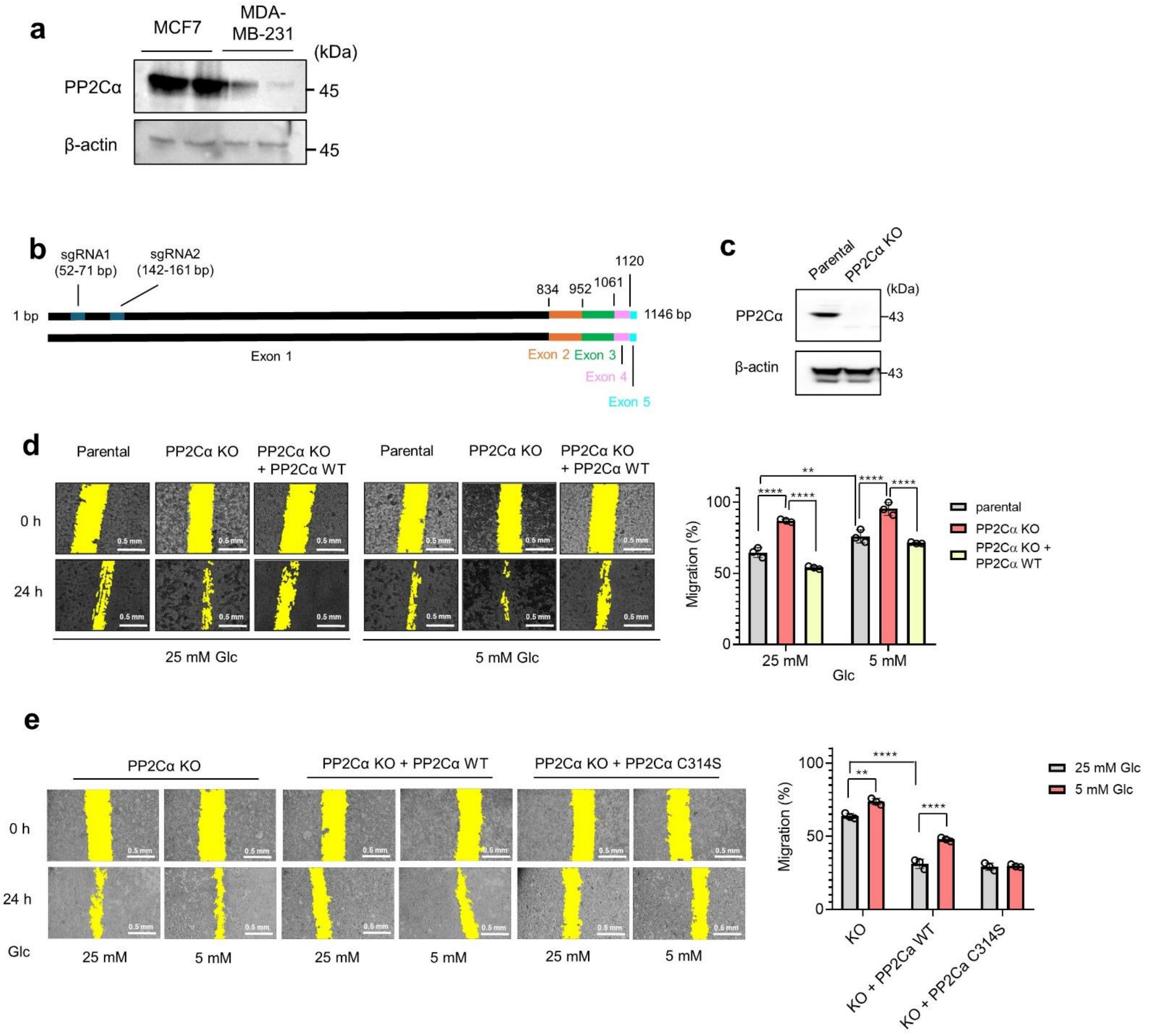

Supplementary Figure 15. Preparation and evaluation of MCF7 PP2Cα KO cell line for cell migration. (**a**) Endogenous PP2Cα expression levels in different cell lines. (**b**) PP2Cα KO gRNA. The positions of two gRNA oligonucleotides are shown in the sequence of PP2Cα. (**c**) PP2Cα expression levels in MCF7 parental and PP2Cα KO cell line. (**d**) The wound healing migration assay of MCF-derived cell line (parental, PP2Cα KO, and PP2Cα KO cell line with the transfection of PP2Cα WT) (n = 3). The scale bar = 0.5 mm. The bar graphs in 25 mM Glc are the same as ones in Figure 5a. (**e**) Migration assay of MCF7-PP2Cα KO expressing PP2Cα WT or C314S in low and high glucose conditions (n = 3). The scale bar = 0.5 mm. The yellow color in (**d-e**) indicates the area without cells. Data represents the mean ± SD. The statistical difference was analyzed by two-way ANOVA and Tukey's post-hoc test, where $*p < 0.03$, $**p < 0.002$, $***p < 0.0002$, $****p < 0.0001$.

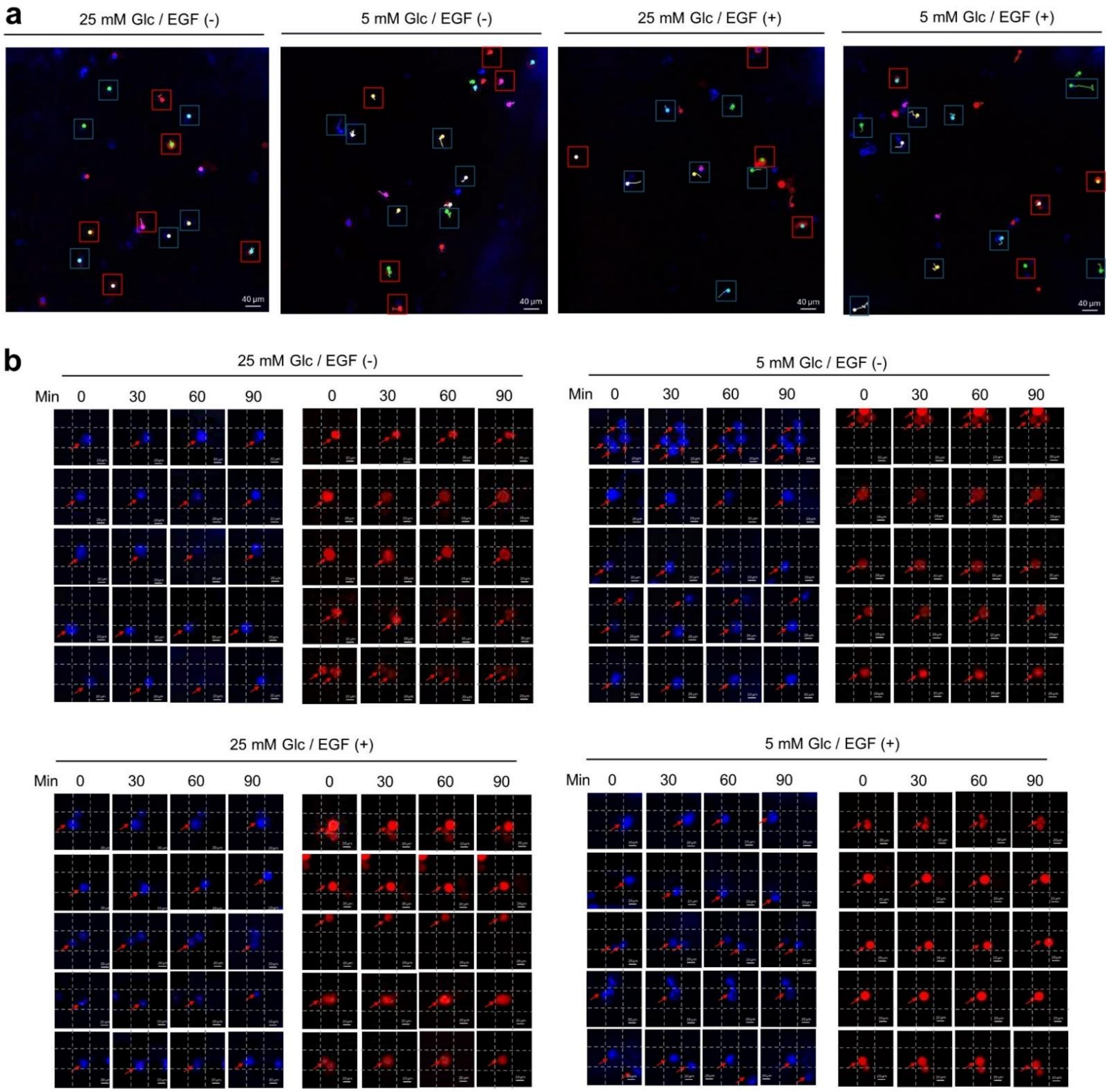

Supplementary Figure 16. Single-cell migration tracking analysis of MCF7 PP2Cα KO cell line. MCF7-PP2Cα KO cells were transfected with PP2Cα WT and Cerulean (MCF7-PP2Cα WT/Cerulean) or PP2Cα C314S and mCherry (MCF7-PP2Cα C314S/mCherry). Two groups of cells were combined and monitored for their migration via fluorescence confocal microscopy. Cells were incubated in low (5 mM) or high glucose (25 mM) without or with EGF for 1.5 h. Images were taken every 10 min and analyzed by Image-J. (**a**) Images showing individual cells with their migration traces. Red cells (mCherry) are boxed with red boxes, whereas blue cells (Cerulean) are boxed with blue boxes. In each box, the migration trace is shown by tails. (**b**) Representative images of 5 cells in blue or red. Images in Figure 5c are re-shown in Figure 16b (quantification analysis is in Figure 5B). The scale bar = 40 μm (**a**) and 20 μm (**b**).

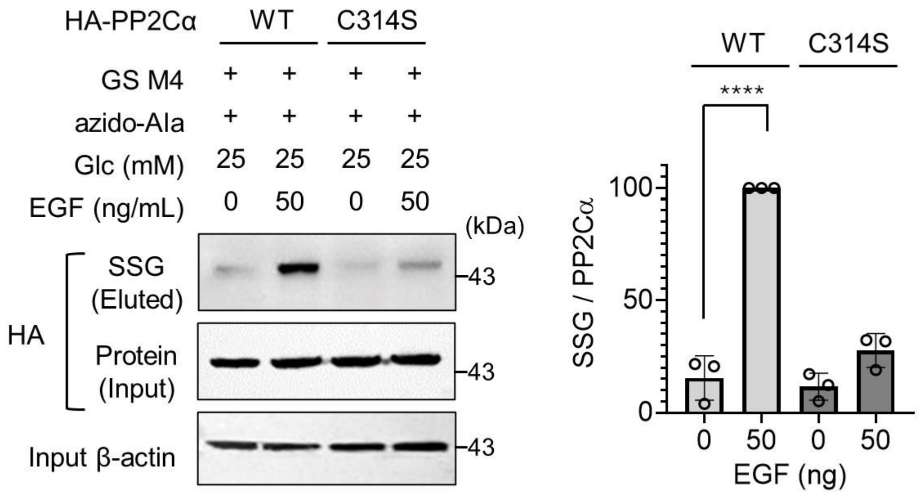

Supplementary Figure 17. EGF induces PP2Cα glutathionylation at C314. MCF7 cells expressing HA-PP2Cα WT or C314S were incubated with EGF for 16 h. Lysates were click-conjugated with biotin-alkyne. Glutathionylated proteins were analyzed by western blot after (SSG level) and before (protein level) enrichment by streptavidin-agarose. Data show the mean ± SD with representative of 3 independent experiments. The statistical difference was analyzed by one-way ANOVA and Tukey's post-hoc test, where *$p < 0.03$, **$p < 0.002$, ***$p < 0.0002$, ****$p < 0.0001$.

Supplementary Methods

Cloning. A human PP2Cα cDNA clone was purchased from OriGene (Cat# SC321896) and subcloned into mammalian vector pcDNA3.1/hygro (+). PCR reaction was performed using a forward primer (5'-GGT GGT GGA TCC GCC ACC ATG GGA GCA TTT TTA GAC AAG C-3') with Kozak sequence and BamHI site and a reverse primer (5'-GGT GGT CTC GAG CTA AGC GTA ATC TGG AAC ATC GTA TGG GTA CCA CAT ATC ATC TGT TGA TGT AGA G-3') with HA tag and XhoI restriction site. The PCR product and the empty pcDNA3.1 vector were double digested with the above restriction enzyme and ligated using T4 DNA ligase to produce pcDNA3.1-HA-PP2Cα. The site-directed mutagenesis was performed using the following primers and pcDNA3.1-HA-PP2Cα as a template. PP2Cα mutant C204S (forward: 5'-GCC CTT GGG GAT TTT GAT TAC AAA AGT GTC CAT GGA AAA GGT CC-3' and reverse: 5'-GGA CCT TTT CCA TGG ACA CTT TTG TAA TCA AAA TCC CCA AGG GC-3'), C238S (forward: 5'-CAG TTC ATT ATC CTT GCA AGT GAT GGT ATC TGG GAT GTT ATG G-3' and reverse: 5- CCA TAA CAT CCC AGA TAC CAT CAC TTG CAA GGA TAA TGA ACT G-3') and C314S (forward: 5'-GGA CAA GTA CCT GGA AAG CAG AGT AGA AGA AAT CAT AAA GAA GC 3' and reverse: 5'-GCT TCT TTA TGA TTT CTT CTA CTC TGC TTT CCA GGT ACT TGT CC-3'). PP2Cα WT and C314S were subcloned into a pET-28a(+) bacterial expression vector using NcoI/XhoI restriction sites. PCR was performed using a forward primer (5'- GGT GGT CC ATG GGA GCA TTT TTA GAC AAG C -3') with a NcoI site and a reverse primer (5'- GGT GGT CTC GAG AGC GTA ATC TGG AAC ATC GTA TGG GTA CCA CAT ATC ATC TGT TGA TGT AGA G -3') with an XhoI site. pcDNA3.1-PP2Cα WT or C314S construct was used as the template for PCR. The PCR product and the empty pET-28a(+) vector were double digested with the above restriction enzymes and ligated using T4 DNA ligase to produce pET28a-PP2Cα WT and C314S plasmids.

PP2Cα WT or C341S mutant with Cerulean or mCherry was cloned into pIRES vector (Takara, Cat# 631605) to produce pIRES-PP2Cα WT-Cerulean and pIRES-PP2Cα C314S-mCherry. PCR for PP2Cα WT or C314S mutant was performed using a forward primer (5'- GGT GGT GCT AGC GCC ACC ATG GGA GCA TTT TTA GAC AAG C-3') with a NheI site, a reverse primer (5'-GGT GGT ACG CGT CTA AGC GTA ATC TGG AAC ATC GTA TGG GTA CCA CAT ATC ATC TGT TGA TGT AGA G-3') with a MluI site. pcDNA3.1-PP2Cα WT and C314S were used as templates for PCR. The PCR for Cerulean or mCherry was performed using a forward primer (5'- GGT GGT TCT AGA ATG GTG AGC AAG GGC GAG-3') with an XbaI site, a reverse primer (5'-

GGT GGT GCG GCC GCC TAC TTG TAC AGC TCG TCC ATG C-3’) with a NotI site. pcDNA3-TORCAR (Addgene, Cat# 64927) or pCMV-mCherry-MHC-IIA (Addgene, Cat #35687) were used as templates for Cerulean and mCherry, respectively. First, PP2Cα WT or C314S mutant was cloned into the pIRES vector by double digesting the PCR and pIRES vector with NheI and MluI, then ligating them with T4 DNA ligase. Subsequently, Cerulean or mCherry was inserted into pIRES-PP2Cα WT or C314S by double digesting the PCR and plasmid with XbaI and NotI, then ligating them using T4 DNA ligase.

To prepare the plasmid for CRISPR knockout for PP2Cα, pSpCas9(BB)-2A-GFP (PX458) plasmid was obtained from Addgene (Cat# 48138). The target region of the gene was analyzed by ENSEMBL (https://useast.ensembl.org/index.html). Using CRISPOR (http://crispor.tefor.net/) and IDT (https://www.idtdna.com/pages), sgRNAs were designed as follows: KO gRNA1 (sense: 5’- CACC G CAGGGTAATGGGTTGCGATA-3’, and antisense: 5’-AAA C TA TCG CAA CCC ATT ACC CTG C-3’) and KO gRNA2 (sense: 5’- CAC CGA CCA CGA TTC AAG TCC ACT-3’, and antisense: 5’- AAA CAG TGG ACT TGA ATC GTG GTC-3’). The sense and antisense primers were annealed by mixing 1 μL of each primer (100 μM), 1 μL of 10x T4 ligase buffer, 1 μL of T4 PNK, and 6 μL of sterile water. The solution was incubated at 37°C for 30 min. The solution was then heated to 95 ^{o}C for 5 min and cooled to room temperature. The annealed primer mixture was diluted at 1:200. The empty plasmid was digested by the BbSI restriction enzyme. The diluted annealed primers and the linear vector were ligated using T4 DNA ligase to produce pSpCas9(BB)-2A-GFP-gRNA1 and pSpCas9(BB)-2A-GFP-gRNA2.

Site-directed mutagenesis. Human JNK plasmid (Cat# 13755), human CSK plasmid (Cat# 74502), and human ARHGEF7 (Cat# 15234) were obtained from Addgene. Mouse MGLL plasmid (Cat# MR204234), mouse RAB21 plasmid (Cat# MR202564), mouse STK10 plasmid (Cat# MR220805), human CFL plasmid (Cat# RC203585), human NISCH plasmid (Cat# RC206026), and human PPM1A plasmid (Cat# SC321896) were obtained from OriGene. Mutants of cysteine to serine were generated by site-directed mutagenesis using the following primers. All cloned plasmids were confirmed by DNA sequencing of the open reading frames.

Mutant protein		Primer sequence 5’ to 3’
JNK C177S	Forward	G GCC CGG ACA GCG TCC ACT AAC TTC ATG ATG

	Reverse	CAT CAT GAA GTT AGT GGA CGC TGT CCG GGC C
CSK C31S	Forward	G GAC CTG CCC TTC TCC AAA GGA GAC GTG C
	Reverse	G CAC GTC TCC TTT GGA GAA GGG CAG GTC C
CSK C290S	Forward	G CTG GGC GGA GAC TCT CTC CTC AAG TTC TC
	Reverse	GA GAA CTT GAG GAG AGA GTC TCC GCC CAG C
ARHGEF7 C490S	Forward	CAA GTC ACC ATT CAG TCT GCG GGA AGC GAG G
	Reverse	C CTC GCT TCC CGC AGA CTG AAT GGT GAC TTG
ARHGEF7 C721S	Forward	GTT ATC GAA GCT TAT TCC ACA AGT GCA AAG AC
	Reverse	GT CTT TGC ACT TGT GGA ATA AGC TTC GAT AAC
MGLL C201S	Forward	CT GAC CCA CTC GTC TCC CGA GCA GGG CTG AAG
	Reverse	CTT CAG CCC TGC TCG GGA GAC GAG TGG GTC AG
MGLL C208S	Forward	CA GGG CTG AAG GTG TCC TTT GGC ATA CAG C
	Reverse	G CTG TAT GCC AAA GGA CAC CTT CAG CCC TG
MGLL C301S	Forward	CA GCA GGA GCT GGG TCT CCA CCC ACG CGT AC
	Reverse	GT ACG CGT GGG TGG AGA CCC AGC TCC TGC TG
RAB21 C27S	Forward	G CTC GGG GAA GGC TCC GTG GGG AAG ACG TCG
	Reverse	CGA CGT CTT CCC CAC GGA GCC TTC CCC GAG C
CFL1 C39S	Forward	G GCG GTG CTC TTC TCC CTG AGT GAG GAC AAG
	Reverse	CTT GTC CTC ACT CAG GGA GAA GAG CAC CGC C
CFL C80S	Forward	G CCA GAT AAG GAC TCC CGC TAT GCC CTC TAT G
	Reverse	C ATA GAG GGC ATA GCG GGA GTC CTT ATC TGG C
NISCH185S	Forward	C CTG GAC TTC ACC TCT CGC CTT AAG TAC C
	Reverse	G GTA CTT AAG GCG AGA GGT GAA GTC CAG G
PP2Cα C314S	Forward	GGA CAA GTA CCT GGA AAG CAG AGT AGA AGA AAT CAT AAA GAA GC
	Reverse	GCT TCT TTA TGA TTT CTT CTA CTC TGC TTT CCA GGT ACT TGT CC

Purification of PP2Cα. C-terminal His-tagged pET28a-PP2Cα WT and C314S mutant were individually expressed and purified in BL21 (DE3) cells. Bacterial expression vectors were transformed into *E. coli* BL21 (DE3) cells. LB medium (10 mL) with kanamycin (50 µg/mL) was inoculated and incubated overnight at 37°C in a rotator. LB medium was diluted into autoclaved 1 L LB medium containing kanamycin. Cells were incubated at 37°C in a shaker until OD_{600} reached 0.8. Cells were then induced with isopropyl-1-thio-β-D-galactopyranoside (IPTG) (0.4 mM) at 37°C for 4 h in the shaker. Cells were harvested by centrifugation at 4,000 rpm for 20 min at 4°C. The cell pellet was resuspended in 20 mL lysis buffer (50 mM Tris-HCl pH 8.3, 300 mM NaCl, 10 mM imidazole, 1 mM DTT, protease inhibitor) and lysed by double passing through a cold French press at 1,000 psi. Cell debris was removed by spinning at 14,000 rpm for 30 min at 4°C, and the supernatant was mixed with prewashed Ni-NTA beads in a purification column for 2 h at 4°C. The lysate was drained, and the beads were washed three times with the washing buffer (12 mL x 3) (50 mM Tris-HCl pH 8.3, 300 mM NaCl, 25 mM imidazole, 1 mM DTT). Proteins were eluted with 10 fractions of 1 mL elution buffer (50 mM Tris-HCl pH 8.3, 300 mM NaCl, 300 mM imidazole, and 1 mM DTT). All the fractions were run on SDS-PAGE to identify the positive fractions. The positive fractions were dialyzed against 50 mM Tris buffer pH 7.4, 10% glycerol, and 1 mM DTT overnight and for 2 h next day with newly prepared dialysis buffer. Proteins were concentrated using a centrifugal filter unit (30 kD cut-off). Protein concentration was measured using Bradford assay with bovine serum albumin (BSA) as a standard.

ROS measurement. Intracellular ROS levels were measured by using 2’,7’-dichlorodihydrofluorescein diacetate (DCF-DA). MDA-MB-231 and MCF-7 cells were maintained in DMEM and seeded into 96-well plates. Next day, cells were incubated with glucose-free DMEM supplemented with different concentrations of glucose (25, 5, and 1 mM) for 24 h. Cells were then incubated with DCF-DA (50 µM) in phenol red-free DMEM with respective glucose concentration for 30 min in the dark. Fluorescence was measured by a microplate reader (BioTek) with excitation (488 nm) and emission (530 nm) wavelengths. Fluorescence intensity was normalized by the fluorescence measured in cells in DMEM with 25 mM glucose, which showed the lowest intensity of fluorescence.

Analysis of PP2Cα cysteine oxidation to sulfinic or sulfonic acid. MDA-MB 231 cells were incubated with glucose-free DMEM supplemented with 5 mM or 25 mM glucose for 12 h and lysed using a RIPA buffer (200 µL)

containing 1% NP-40, 0.25% sodium deoxycholate, 150 mM NaCl, 1 mM EDTA, 50 mM Tris (pH 8.0), and a protease inhibitor cocktail. As a positive control, the lysate of MDA-MB 231 cells grown in DMEM with 25 mM glucose was treated with 10 mM H_2O_2, followed by 25 mM IAM (Iodoacetamide) for 15 min. As a negative control, the lysate of MDA-MB 231 cells grown in DMEM with 25 mM glucose was treated with 25 mM IAM, followed by 10 mM H_2O_2. Lysates (1 mg) were used for the immunoprecipitation of HA-PP2Cα by using HA antibody. The western blots were carried out using the HA- and cysteine (sulfonate, SO_3H)- antibodies (1:1000; Enzo LifeSciences, Cat# ADIOSA820D).

Antibodies validation. Antibodies were used as purchased from the vendors (JNK, p-JNK, p38, p-p38, MEK1/2, p-MEK1/2, p65, p-p65, MKK4, p-MKK4, p-PAK1, PAK1, p44/42, p-44/42, SMAD3, p-SMAD3, actin) or validated by the overexpression or knockout of target genes (HA, PP2Cα, SO_3H).
